# Semilunar Granule Cells Are the Primary Source of the Perisomatic Excitatory Innervation onto Parvalbumin-Expressing Interneurons in the Dentate Gyrus

**DOI:** 10.1523/ENEURO.0323-19.2020

**Published:** 2020-07-07

**Authors:** Laura Rovira-Esteban, Norbert Hájos, Gergő Attila Nagy, Carlos Crespo, Juan Nacher, Emilio Varea, José Miguel Blasco-Ibáñez

**Affiliations:** 1 Department of Cell Biology, Neurobiology Unit, Interdisciplinary Research Structure for Biotechnology and Biomedicine (BIOTECMED), University of Valencia, E-46100 Burjasot (Valencia), Calle Dr Moliner 50 Valencia, Spain; 2 Institute of Experimental Medicine, Hungarian Academy of Sciences, H-1083 Budapest, Szigony utca 43 Hungary; 3 János Szentágothai Doctoral School of Neurosciences, Semmelweis University, H-1085 Budapest, Üllői út 26 Hungary; 4 Spanish National Network for Research in Mental Health (CIBERSAM), E-28029, Madrid, Avenida Monforte de Lemos 3-5 Pabellón 11, Planta 0, Spain

**Keywords:** dentate gyrus, electron microscopy, immunochemistry, microcircuitry, tracing

## Abstract

We analyzed the origin and relevance of the perisomatic excitatory inputs on the parvalbumin interneurons of the granule cell layer in mouse. Confocal analysis of the glutamatergic innervation showed that it represents ∼50% of the perisomatic synapses that parvalbumin cells receive. This excitatory input may originate from granule cell collaterals, the mossy cells, or even supramammillary nucleus. First, we assessed the input from the mossy cells on parvalbumin interneurons. Axon terminals of mossy cells were visualized by their calretinin content. Using multicolor confocal microscopy, we observed that less than 10% of perisomatic excitatory innervation of parvalbumin cells could originate from mossy cells. Correlative light and electron microscopy revealed that innervation from mossy cells, although present, was indeed infrequent, except for those parvalbumin cells whose somata were located in the inner molecular layer. Second, we investigated the potential input from supramammillary nucleus on parvalbumin cell somata using anterograde tracing or immunocytochemistry against vesicular glutamate transporter 2 (VGLUT2) and found only occasional contacts. Third, we intracellularly filled dentate granule cells in acute slice preparations using whole-cell recording and examined whether their axon collaterals target parvalbumin interneurons. We found that typical granule cells do not innervate the perisomatic region of these GABAergic cells. In sharp contrast, semilunar granule cells (SGCs), a scarce granule cell subtype often contacted the parvalbumin cell soma and proximal dendrites. Our data, therefore, show that perisomatic excitatory drive of parvalbumin interneurons in the granular layer of the dentate gyrus is abundant and originates primarily from SGCs.

## Significance Statement

The microcircuitry underlying each cerebral structure is important to understand its function in normal and pathologic conditions. We analyzed the excitatory innervation on the soma of the parvalbumin-expressing interneurons in the dentate gyrus. Excitatory innervation on the soma is in a privileged position to drive more efficiently the spiking of neurons than that on dendrites. How parvalbumin interneurons are recruited is crucial for a proper knowledge of the function that they play in dentate gyrus operation. Our results show that the main source of this innervation are the semilunar granule cells (SGCs), a rare subtype of granule cells. Thus, this scarce excitatory cell type of the dentate gyrus is in a strategic position to effectively control network operation via feed-forward inhibition.

## Introduction

The general connectivity of the dentate gyrus has been well described, but details on several cell types have not been completely elucidated. The principal cells of the dentate gyrus, the granule cells, are located in the granule cell layer where their somata are densely packed. The axons of the granule cells, the mossy fibers, project to the CA3 subfield of the hippocampus through the hilus, where they contact the mossy cells, a population of excitatory cells that project back to the granule cells forming an excitatory loop (Ribak et al., 1985; Blasco-Ibáñez and Freund, 1997). The granule cell dendrites ramify in the molecular layer, which can be divided into the inner molecular layer, that hosts the projection from the mossy cells and the supramammillary nucleus, and the middle and outer molecular layers, both receiving the fibers from the entorhinal cortex.

Dispersed in the molecular layer, granule cell layer and hilus, there are different populations of interneurons (Freund and Buzsáki, 1996). Among them, parvalbumin interneurons belong to two populations of GABAergic fast-spiking cells that form either axo-somatic (basket cells) or axo-axonic (chandelier cells) symmetric inhibitory synapses on granule cells (Kosaka et al., 1987; Soriano and Frotscher, 1989; Nitsch et al., 1990; Soriano et al., 1990; Han et al., 1993; Howard et al., 2005; Freund and Katona, 2007) . The somata of these GABAergic cells are located predominantly in the granule cell layer and inner molecular layer. Parvalbumin interneurons in the dentate gyrus have various morphologies (Ribak and Seress, 1983). Most of them display pyramidal morphology with their somata sitting in the infragranular layer, whereas others have their somata in the supragranular layer or in the inner molecular layer.

Parvalbumin basket cells of the dentate gyrus are considered key elements for the normal and pathologic function of this brain structure (Sloviter et al., 2003). In addition to inhibitory inputs on their soma and apical dendrite, parvalbumin interneurons receive a rich perisomatic innervation from axon terminals forming asymmetric synapses (Ribak and Seress, 1983). However, neither the relative abundance of the excitatory inputs on the perisomatic region, nor their origin have been elucidated.

Fibers containing high concentrations of synaptic zinc, likely arising from granule cells, have been described to contact heavily the perisomatic region of parvalbumin cells in rats (Blasco-Ibáñez et al., 2000), although the particular population of cells from which this projection originates has not been identified yet. In addition, mossy cell axons from the hilus projecting to the inner molecular layer through the granule cell layer have been considered to be an important contributor to the perisomatic asymmetric input on the parvalbumin basket cells (Seress and Ribak, 1984). Finally, afferents from the supramammillary nucleus have been described to contact perisomatically at least some parvalbumin interneurons in the dentate gyrus of the monkey (Leranth and Nitsch, 1994; Nitsch and Leranth, 1994). Thus, all these three major excitatory inputs may innervate parvalbumin interneurons perisomatically, but it is still unknown to what extent.

Granule cells have been considered a homogeneous population regarding connectivity and functionality. Recently, a subpopulation of granule cells first described morphologically as semilunar granule cells (SGCs) by Ramón y Cajal (1904) has been further analyzed. These SGCs have different morphology than classical granule cells: they are multipolar instead of monopolar cells (in rodents), and their dendritic arbor extends more widely in the molecular layer. They are located prevalently near the supragranular layer or deeper in the inner molecular layer. SGCs have a special connectivity with mossy cells, as these hilar excitatory cells can be engaged by this special granule cell type during sustained recurrent excitation known as hilar up-states (Williams et al., 2007; Larimer and Strowbridge, 2010). Hilar up-states are not only maintained by the recurrent connectivity between these cells, but also by the firing characteristics of SGCs. Interestingly, and in contrast to typical granule cells, the axon of SGCs may extend collaterals within the granule cell layer (Williams et al., 2007). These morphologic findings suggest that this particular population of granule cells is in an appropriate position to be a source of the perisomatic zincergic boutons on parvalbumin interneurons.

In the present work, we aim to reveal the source and relevance of the excitatory perisomatic innervation of parvalbumin interneurons in the dentate gyrus as well as to test specifically whether SGCs provide the zincergic projection on these GABAergic cells.

## Materials and Methods

### Animals

CD-1 ICR mice (Harlan) were used for this work. Animals were housed in groups of three to six under controlled temperature, humidity, and on a 12/12 h light/dark cycle, with access to food and water *ad libitum*. They were allowed to habituate to our facilities at least 2 d before the experiments. An effort was made to avoid unnecessary stress in the animals due to handling. All animal experimentation was conducted in accordance with the Directive 2010/63/EU of the European Parliament and of the European Council of 22 September 2010 on the protection of animals used for scientific purposes.

Adult animals included in this study were between two and five months old. No differentiation was made between males and females as there is no evidence of anatomic differences between them in the expression of any of the studied cell markers, or in the studied projections. Animals were perfused transcardially under deep pentobarbital anesthesia, first with 10 ml of physiological saline followed by 30 min of fixative, at a flow rate of 4 ml/min. A fixative containing 4% paraformaldehyde (PFA) with 15% of a saturated solution of picric acid in 0.1 m phosphate buffer (PB) was used for light microscopy and confocal studies, whereas for electron microscopy the fixative contained 0.5% glutaraldehyde in addition. For Timm staining, the animals were initially perfused with 20 ml of 0.05% Na_2_S in PB instead of saline (4 ml/min), followed by the fixative for light microscopy. After perfusion, brains were removed and cut as 60-μm-thick coronal sections on a vibratome (VT1000S, Leica). All the tissue was stored in PB with 0.05% sodium azide at 4°C.

### Tracer injection

Four adult mice (approximately three to four months old) were anesthetized and then, using a stereotaxic apparatus (Kopf Instruments), were intracerebrally injected with the anterograde tracer biotin dextran amine (BDA; 10 kDa; 10% in 0.1 m PB; Invitrogen) in the supramammillary nucleus (bregma −2.80 mm, lateral 0 mm, depth 4.3 mm). Coordinates were calculated according to the Paxinos’ Atlas (Paxinos and Franklin, 2012). The tracer was delivered by iontophoresis via 7-s on/off 5- to 10-μA current pulses for 15 min using a borosilicate micropipette. After 5–7 d of survival, animals were transcardially perfused with the glutaraldehyde fixative as described previously. A parallel series of sections taken from each animal (one in six sections) was incubated in avidin-biotin complex (ABC; 1:200, DAKO) in PB containing 0.1% Triton X-100 and developed using nickel intensified diaminobenzidine (DAB) to visualize the injection site and the anterograde labeling.

### Fluorescent immunohistochemistry

The immunostaining for confocal microscopy was done in free floating sections. Sections were blocked using 10% normal donkey serum (NDS) in PB saline containing 0.2% Triton X-100 (PBS-Tx) for 1 h at room temperature. The incubations with the primary antibodies were performed overnight at room temperature, or 48 h at 4°C. The following primaries were used: mouse anti-bassoon (1:2000; ab82958 Abcam), rabbit anti-calretinin (1:2000; 7699/3H Swant), mouse anti-calretinin (1:5000; 6B3 Swant), guinea pig anti-parvalbumin (1:2000; 195004 Synaptic Systems), rabbit anti-parvalbumin (1:5000; PV-28 Swant), goat anti-PSD95 (1:1000; ab12093 Abcam), mouse anti-gephyrin (1:1000; 147011 Synaptic Systems), and mouse anti-synaptophysin (1:1000; S5768 Sigma). The secondary antibodies (1:400) were donkey anti-rabbit IgG conjugated with Alexa Fluor 488, Alexa Fluor 555, or Alexa Fluor 647 (Invitrogen); donkey anti-mouse IgG conjugated with Alexa Fluor 488, DyLight 549 or 649 (Jackson ImmunoResearch); goat anti-guinea pig IgG conjugated with Alexa Fluor 488 (Invitrogen); and donkey anti-guinea pig IgG conjugated with DyLight 549 or 649 (Jackson ImmunoResearch). Mounting Medium for fluorescence (refractive index 1.47–1.5; DAKO) was used to mount the section onto the slides. Slides were analyzed under a confocal scanning microscope (Leica TSC-SPE). For quantification of the perisomatic innervation of parvalbumin cells, Z-series of focal planes were taken from the surface of the section to the maximal Z that penetration of the antibody allowed (HCX PL APO 63× oil objective; NA: 1.4, Z step size 0.5 μm, *xy*: 0.07 μm/pixel). For image analysis we used FIJI (ImageJ 1.49j10; Schindelin et al., 2012). Appositions of synaptic puncta were quantified as follows: for presynaptic markers, synaptophysin, calretinin, bassoon, and VGluT2; only those puncta that overlapped the outside edge of the parvalbumin profiles with no apparent space between both profiles were considered as positive; for postsynaptic markers, PSD95 and gephyrin; only those puncta inside the parvalbumin profile near the inner edge of the parvalbumin profile were considered as positive. In the study comparing the excitatory and inhibitory inputs to the perisomatic area of parvalbumin interneurons, data are given as percentages of PSD95 or gephyrin in relation to the total number of puncta expressing postsynaptic markers, which was calculated as the sum of PSD95-positive elements and gephyrin-positive elements in close apposition to the soma border by its inner side.

### Immunohistochemistry for electron microscopy

For electron microscopy, we used sections from animals fixed with a fixative containing glutaraldehyde. To enhance the penetration of the antisera, the sections were cryoprotected in 10% glycerol, 25% sucrose in 0.01 m PB and then freeze-thawed three times above liquid nitrogen. No detergent was used either for washing or incubations. The sections were then treated in 1% sodium borohydride in 0.1 m PB for 20 min. Afterwards, they were blocked in 10% normal goat serum dissolved in 0.1 m PB. Then, the sections were incubated with mouse anti-calretinin (1:5000; 6B3 Swant) for 2 d at 4°C. As secondary antibody, we used biotinylated goat anti-mouse IgG (1:200; Invitrogen) for 2 h. Then, we used the ABC method (1:200; DAKO) and developed the color using the Ni-intensified DAB technique (black color). After that, the sections were subjected to a second immunostaining using rabbit anti-parvalbumin (1:5000; PV-28 Swant), biotinylated goat anti-rabbit IgG (1:200; Invitrogen), and ABC (1:200; DAKO). Subsequently color was developed using DAB, which produces brown color. Double stained sections were treated with 1% OsO_4_ (Electron Microscopy Sciences) and 7% glucose in 0.1 m PB for 45 min at room temperature. Then, sections were treated with 2% uranyl acetate in maleate buffer. Afterwards, they were dehydrated in ascending series of cold ethanol, cleared in propylene oxide, embedded in epoxy resin (Durcupan, Sigma), and flat mounted on microscope slides. Specimens were studied under the light microscope, and the cells to be further analyzed using electron microscopy were isolated and re-embedded on an epoxy resin block. Then, 50-nm-thick ultrathin sections were obtained using an ultramicrotome (Leica EM UC6), collected on Formvar-coated grids and counterstained with lead citrate for 12 min. Sections were examined using a JEOL JEM-1010 transmission electron microscope. Images were acquired using either MegaView I digital camera or AMT RX80 digital camera. Serial ultrathin sections were collected on Formvar-coated slot grids until the penetration of the calretinin antibody diminished. All consecutive ultrathin sections were analyzed, to verify whether the calretinin-containing profiles made synaptic contact on the cells.

### Synaptic zinc histochemistry

Animals destined to zinc histochemistry were processed for Timm staining immediately, to avoid the loss of zincergic fibers. Sections were washed 3 × 20 min in 0.1 m PB and then incubated in a solution containing: 14% acacia gum, 1.7% hydroquinone, 0.08% silver nitrate, 2.4% citric acid, and 2.3% trisodium citrate. Autometallography was stopped using 5% sodium thiosulfate in PB. Then, sections were processed for immunohistochemistry against parvalbumin (rabbit anti-parvalbumin, 1:5000; PV-28 Swant) using the ABC method or mounted directly.

### Whole-cell patch-clamp

For the whole-cell patch-clamp recordings, postnatal days 15-23 CD-1 mice were used. This range was chosen because it allowed a high survival of cells when preparing slices, with a relatively well-formed dentate gyrus. In addition, this age also ensured that the possible postsynaptic parvalbumin interneurons would remain as healthy as possible and were not degenerating in the slice. Pups were deeply anesthetized with isoflurane (IsoFlo 1385 ESP, Esteve Veterinaria) and decapitated. The head was immediately transferred into an ice-cold solution containing the following: 2.5 mm KCl, 5 mm MgCl_2_, 0.5 mm CaCl_2_, 1.25 mm NaH_2_PO_4_, 10 mm glucose, 26 mm NaHCO_3_, and 252 mm sucrose, bubbled with carbogen (95% O_2_/5% CO_2_), and then the brain was quickly removed from the skull. Horizontal slices of 300-μm thickness were cut using a vibratome (VT 1000S, Leica) and placed into an interface-type holding chamber containing artificial CSF (aCSF) at room temperature. aCSF contained the following: 2.5 mm KCl, 2 mm MgCl_2_, 2 mm CaCl_2_, 1.25 NaH_2_PO_4_ mm, 10 mm glucose, 26 mm NaHCO_3_, and 126 mm NaCl, bubbled with carbogen. Sections were stabilized in this solution for at least 1 h before placing them into a recording chamber.

Patch pipettes were pulled from borosilicate glass capillaries with 1.5 mm outer diameter and 0.84 mm inner diameter (World Precision Instruments) using a P-97 puller (Sutter Instruments). They were filled with a solution that contained the following: 4 mm NaCl, 110 mm K-gluconate, 20 mm KCl, and 10 mm HEPES, and 2 mm MgCl_2_. Biocytin (Sigma) was included in the pipette solution to procure the intracellular labeling of the recorded cell. Pipette resistance was checked in the bath, varying from 3 to 6 MΩ. Whole-cell recordings were performed in oxygenated aCSF under visual guidance using an Olympus Microscope (BX51WI, Olympus) with differential interference contrast optics, visualized by a NIR CCD camera (C7500-51 Hamamatsu). The amplifier used was an AM2400 (AM Systems), connected to a CED Micro 1401 AD converter (Cambridge Electronic Design Limited). Recorded neurons were kept for 5 min in Vclamp at a holding voltage of −75 mV, as it has been described that these cells have a hyperpolarized resting membrane potential (Spruston and Johnston, 1992; Staley et al., 1992; Penttonen et al., 1997; Williams et al., 2007). Patched cells were kept firing action potentials for at least 10 min to improve the axonal filling. After 10–20 min, the slices were fixed by immersion in a fixative solution containing 4% PFA and 0.5% glutaraldehyde. Fixed slices were re-sectioned to 60-μm-thick sections and then processed for biocytin detection using ABC (1:200, 4 h). The reaction was visualized using Ni-intensified DAB. Those cells that presented collaterals with varicosities in the inner molecular layer and granule cell layer were further processed for parvalbumin immunoreactivity (rabbit anti-parvalbumin, 1:5000; PV-28 Swant) using DAB, either for light or electron microscopy as described above. A subset of the fixed slices was developed with Alexa Fluor 488-conjugated streptavidin (1:400; Molecular Probes S11223) in PB containing 0.2% Triton X-100, mounted in DAKO fluorescent mounting medium and imaged using a confocal scanning microscope (Leica TSC-SPE, HCX PL APO U-V-I 40× objective; NA: 0.75, Z step size 0.84 μm, *xy*: 0.27 μm/pixel). Once imaged, slices containing collaterals with varicosities in the granule cell layer were demounted, re-sectioned to 60-μm-thick sections, developed using Ni-intensified DAB, and processed for parvalbumin immunoreactivity as described previously. An additional batch of slices was developed with Cy3-conjugated streptavidin (1:5000; Sigma-Aldrich S6402) in PB containing 0.5% Triton X-100, and subsequently immunostained for parvalbumin [rabbit anti-parvalbumin (1:1000; SySy 195002) in PB containing 1% Triton X-100, and visualized using an A488-conjugated donkey anti-rabbit antibody (1:400; Jackson 711545152).

### Morphometric analysis of granular and SGCs

To analyze the morphologic differences between both populations of granule cells, we used confocal stacks of intracellularly filled cells visualized using Streptavidin-A488 or Streptavidin-Cy3. We performed the Sholl analysis of the dendritic tree (Sholl, 1953), using the Stitching plugin and the Simple Neurite Tracer plugin in ImageJ. We also evaluated the number of primary dendrites, dendritic spread angle (considering a vertex the most basal part of the soma and the sides as the most lateral dendrites in the outer molecular layer), total dendritic length and horizontal diameter of the somata.

### Statistical analysis

Statistical analysis was performed using OriginPro2015. For comparison of the proportion of PSD95 and gephyrin, a *t* test with no assumption of equal variances was used against the hypothesis of the proportion of PSD95 being higher than 50%. For the comparison of the morphometric analysis, the Mann–Whitney test was used. Graphics were produced by using OriginPro 2015. Data are shown as mean ± SEM.

## Results

### Perisomatic excitatory innervation on parvalbumin-expressing interneurons in the granule cell layer

**Figure 1.**
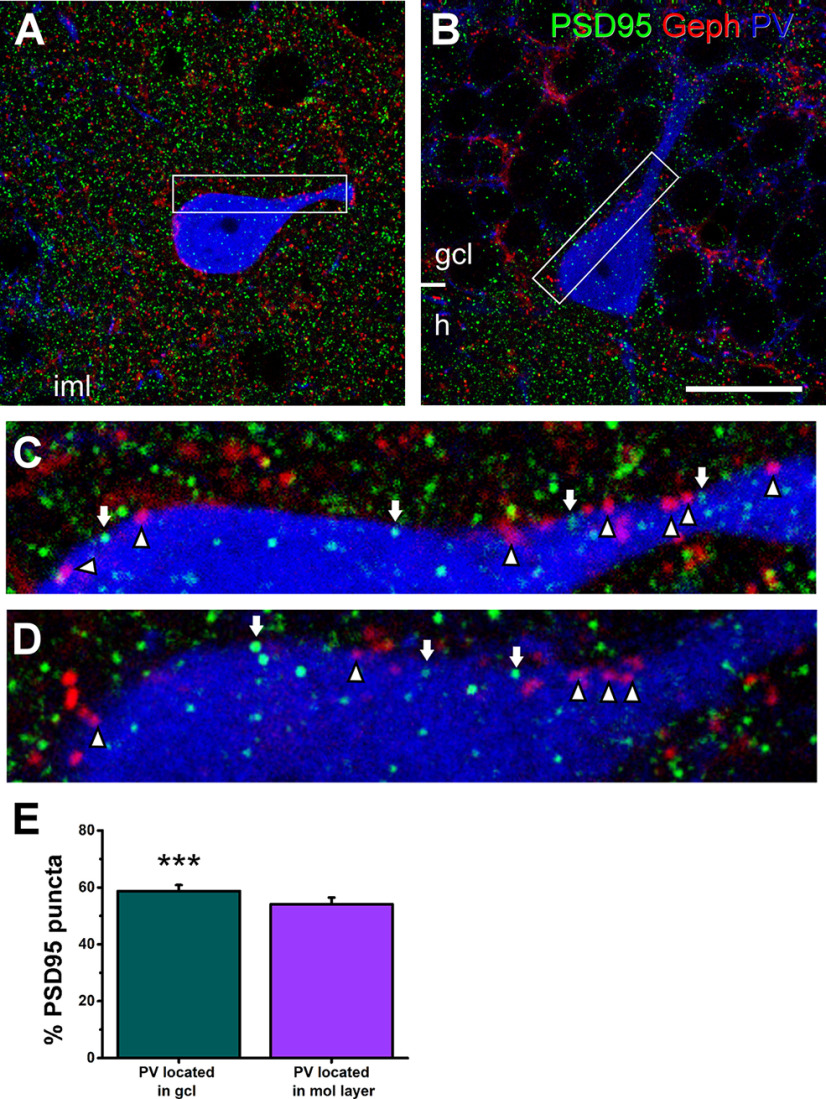
The excitatory and inhibitory perisomatic innervation on parvalbumin interneurons in the dentate gyrus. Triple immunostaining for parvalbumin (blue), gephyrin (red), and PSD95 (green) postsynaptic markers for inhibitory and excitatory synapses, respectively. ***A***, Confocal image showing the soma and a proximal dendrite of a parvalbumin interneuron located in the inner molecular layer (iml). Note the high density of PSD95 puncta in this area. ***B***, Confocal image showing the soma and dendritic trunk of a parvalbumin interneuron with the cell body sitting at the border between the granule cell layer (gcl) and the hilus (h), and the dendritic trunk traveling toward the molecular layer. As expected, the labeling of PSD95 was scarce in the granule cell layer but abundant in the h. ***C***, Higher magnification of the inset shown in ***A***. The number of gephyrin (arrowheads) and PSD95 puncta (arrows) was approximately even for all the cells analyzed. ***D***, Higher magnification of the inset shown in ***B***. The number of gephyrin (arrowheads) at the parvalbumin interneuron surface is lower than PSD95 puncta (arrows) for all the cells analyzed. ***E***, Graph showing the percentage of PSD95 puncta on the sampled parvalbumin cells located either in the granule cell layer or in the inner molecular layer. Vertical bars represent SEM. Asterisks show significance of the statistical analysis for the predominance of PSD95 puncta; ***p < 0.001. Geph, gephyrin; PV, parvalbumin. Scale bar: 20 μm.

To assess the ratio of perisomatic excitatory innervation received by parvalbumin interneurons, multicolor immunofluorescence microscopy was performed. We combined parvalbumin immunostaining with the visualization of the following postsynaptic markers: postsynaptic density protein 95 kDa (PSD95) and gephyrin (Fig. 1). PSD95 is located in the postsynaptic density at asymmetric synapses and functions as an anchoring element for AMPA and NMDA receptors among other synaptic proteins (Lin et al., 2004; Jackson and Nicoll, 2011). Gephyrin labels specifically the postsynaptic specialization of inhibitory synapses (Fritschy et al., 2008).

Using a triple immunostaining for parvalbumin, PSD95 and gephyrin, a total of 16 molecular layer parvalbumin interneurons (Fig. 1*A*) and 16 parvalbumin interneurons sitting in the granule cell layer (Fig. 1*B*) were analyzed in the dentate gyrus of three different animals. The cell depth analyzed in each parvalbumin cell was the same for both postsynaptic density markers (average of 9 μm). Approximately 200 positive elements were quantified per cell, in the soma and proximal portion of the dendrites, which has been considered functionally equivalent to the soma (Gulyás et al., 1999; Megías et al., 2001; Papp et al., 2001; Vereczki et al., 2016). Molecular layer parvalbumin interneurons had about the same number of PSD95-positive as gephyrin-positive puncta (1709 and 1280 puncta in 16 cells; 54.03 ± 2.39% and 45.97 ± 2.40%; *p* > 0.05; Fig. 1*A*,*C*). Granule cell layer parvalbumin cells presented more PSD95-positive than gephyrin-positive puncta (1962 and 1329 puncta in 16 cells; 58.72 ± 2.12% and 41.28 ± 2.12%, respectively; *p* < 0.001; Fig. 1*B*,*D*). For the total number of parvalbumin cells, the percentage of PSD95-positive postsynaptic elements was also higher than the percentage of gephyrin-positive postsynaptic elements (3617 and 2609 puncta in 32 cells; 56.38 ± 1.62% and 43.62 ± 1.62%, respectively; *p* < 0.001).

In summary, our results showed that parvalbumin cells presented slightly more PSD95-positive elements than gephyrin-positive ones. Therefore, the number of perisomatic excitatory boutons on parvalbumin interneurons is higher than the number of inhibitory ones.

### Mossy cells contribute only marginally to the perisomatic excitatory innervation of parvalbumin interneurons in the granule cell layer

As synaptic contacts from the hilar mossy cell axons in their way into the inner molecular layer have been described among the possible sources of perisomatic excitatory connections on parvalbumin interneurons (Seress and Ribak, 1984), we aimed to assess their abundance. To this end, we analyzed the perisomatic innervation of parvalbumin interneurons in the granule cell layer using cellular and synaptic markers and studied their colocalization at the confocal microscopic level.

**Figure 2.**
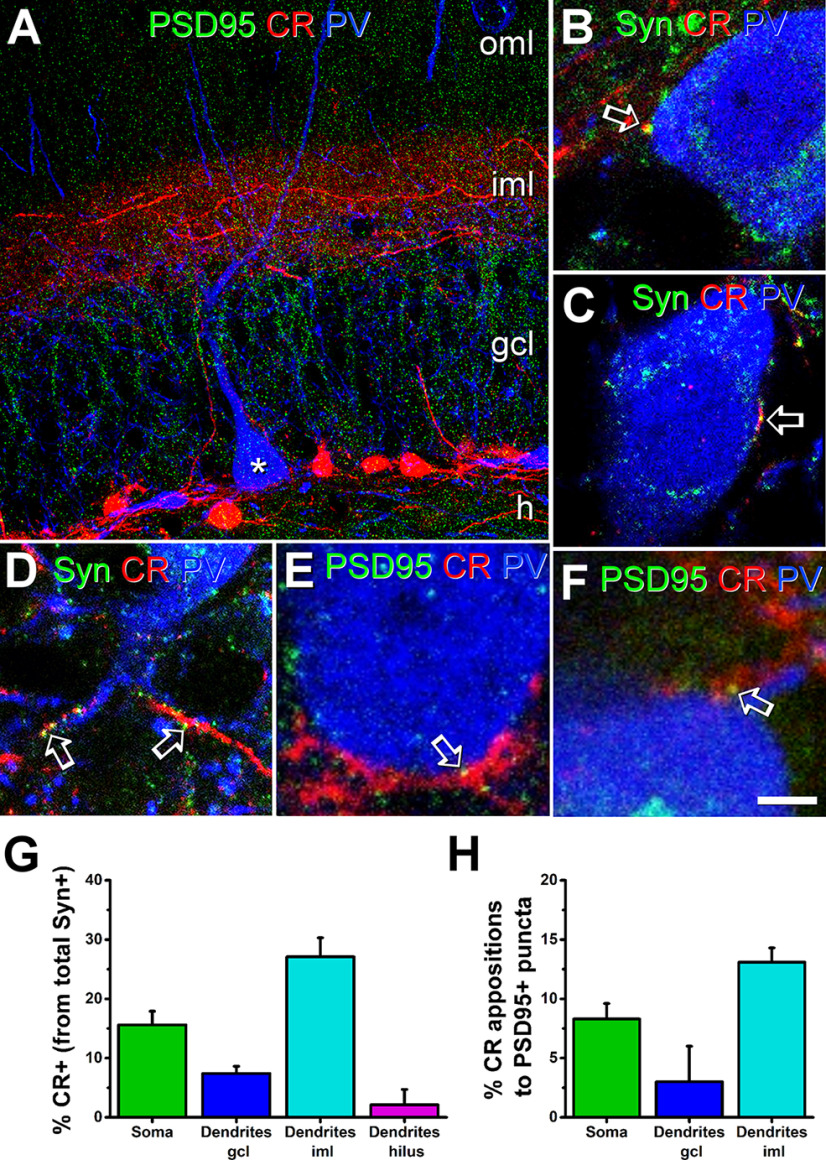
Perisomatic innervation of parvalbumin interneurons by mossy cells detected by confocal microscopy. Confocal analysis of the calretinin-positive elements (red) found in apposition to parvalbumin-positive interneurons (blue) and their co-expression of synaptic markers (green). ***A***, Maximal intensity projection image taken from a parvalbumin interneuron in the granule cell layer near to calretinin-expressing fibers of mossy cells that run through the granule cell layer (gcl) and sprout densely into the inner molecular layer (iml). ***B***–***D***, Colocalization of synaptophysin (green) and calretinin (red) elements in close apposition to somata (***B***, ***C***) and inner molecular layer dendrites (***D***) of different parvalbumin-positive cells (blue). The presence of double immunoreactive elements on parvalbumin cells (open arrows) indicates that some fibers from mossy cells can establish synaptic contacts with parvalbumin cells. ***E***, ***F***, Colocalization of PSD95 (green) and calretinin (red) puncta in close apposition to the soma (***E***) and to a proximal dendrite (***F***) of a parvalbumin-positive interneuron (blue). Although rarely, some apposition of calretinin and PSD95 can be found on parvalbumin somata (open arrows), indicating potential perisomatic contacts of mossy cells with parvalbumin cells. ***G***, ***H***, Graphs showing the proportion of synaptophysin puncta that were calretinin immunoreactive in apposition to parvalbumin elements (***G***) and the proportion calretinin immunoreactive boutons facing PSD95 puncta in apposition to parvalbumin elements (***H***) sampled on the somata and dendrites in the hilus, granule cell layer, and inner molecular layer. Vertical bars represent SEM CR, calretinin; h, hilus; oml, outer molecular layer; PV, parvalbumin; Syn, synaptophysin. Scale bar: 20 μm (***A***) and 5 μm (***B–F***).

To specifically label the mossy cell axons, we used calretinin, as it has been proved to reliably stain mossy cell fibers and synaptic boutons in the mouse (Blasco-Ibáñez and Freund, 1997), whereas parvalbumin was used as a marker for fast-spiking interneurons. Labeling for calretinin and parvalbumin was combined with different synaptic markers. Synaptophysin was used as a marker of the presynaptic elements and PSD95 to visualize the postsynaptic elements of excitatory contacts and to avoid considering calretinin-containing GABAergic axons. A total of six parvalbumin cells from three different animals, selected by their morphology and dendritic distribution, were analyzed to quantify the presence of calretinin-expressing axonal varicosities expressing synaptic markers in close apposition to parvalbumin interneurons (Fig. 2*A*). We only considered for quantification those parvalbumin interneurons located in the granule cell layer.

Triple immunohistochemistry with parvalbumin, calretinin, and synaptophysin antibodies (Fig. 2*B–D*) allowed us to estimate the maximum possible number of calretinin-positive boutons from mossy cells targeting parvalbumin interneurons. Our results showed that mossy cells may perisomatically innervate parvalbumin interneurons, although less than expected. First, we analyzed 294 synaptophysin-positive puncta in close apposition to parvalbumin cells, of which 43 (14.6%) were located on the soma, 85 (28.9%) were located on the granule cell layer dendritic shafts, 135 (45.9%) on the dendrites located in the inner molecular layer, and 31 (10.6%) boutons on proximal dendrites located in the hilus. When these synaptophysin-containing profiles were checked for colocalization with calretinin, we found that the presence of putative synaptic boutons in apposition with parvalbumin cells is more evident in the inner molecular layer, were mossy cells send their axon terminals (27 ± 3% of synaptophysin-positive elements in apposition with parvalbumin dendritic trunks in this area contained calretinin). On the soma, they were less abundant, although we also found some double labeled puncta (16 ± 3%). On the parvalbumin-positive dendrites located in the granule cell layer, we only found some calretinin-positive presynaptic elements (7 ± 2%). Finally, in the dendrites located in the subgranular region of the dentate gyrus, we rarely found calretinin-positive presynaptic elements (2 ± 3%) in close apposition to a parvalbumin-containing profile.

However, this approach had two limitations. First, it did not show whether the synaptophysin-containing boutons correspond to synaptic contacts with the interneuron. Second, it did not allow us to distinguish between GABAergic and glutamatergic boutons, as both may express calretinin. To overcome these limitations, we next made a triple immunohistochemistry with PSD95, calretinin, and parvalbumin and analyzed how many calretinin-positive puncta were adjacent to PSD95 labeling at the membrane surface of parvalbumin cells (Fig. 2*E*,*F*). To this end, we analyzed six cells and quantified a total of 281 PSD95 puncta located in the inner side of the cell surface. PSD95 puncta were abundant on somata (*n* = 104) but only 8 ± 1% were close to calretinin-immunoreactive elements. On the dendrites in the granule cell layer, even less (3 ± 3%, *n* = 18) PSD95 puncta apposed calretinin-containing profiles. Colocalization was higher in the inner molecular layer dendrites, where 13 ± 1% of the PSD95 puncta were juxtaposed to calretinin elements (*n* = 125). None of the 34 PSD95 puncta on parvalbumin dendrites in the hilus was attributable to calretinin elements.

Comparison of these two independent datasets obtained by immunostaining for synaptophysin and PSD95 indicates that the number of calretinin-expressing boutons apposed to PSD95 on the parvalbumin interneuron surface was only a small fraction of the number of calretinin-expressing axon terminals containing synaptophysin. Several reasons may account for these observations, including (1) a part of the boutons containing calretinin and synaptophysin could correspond to GABAergic axon terminals; (2) some calretinin-positive glutamatergic varicosities do not form synapses; and (3) a few calretinin-containing mossy cell terminals are so small that the detection of PSD95 at these boutons may be compromised.

**Figure 3.**
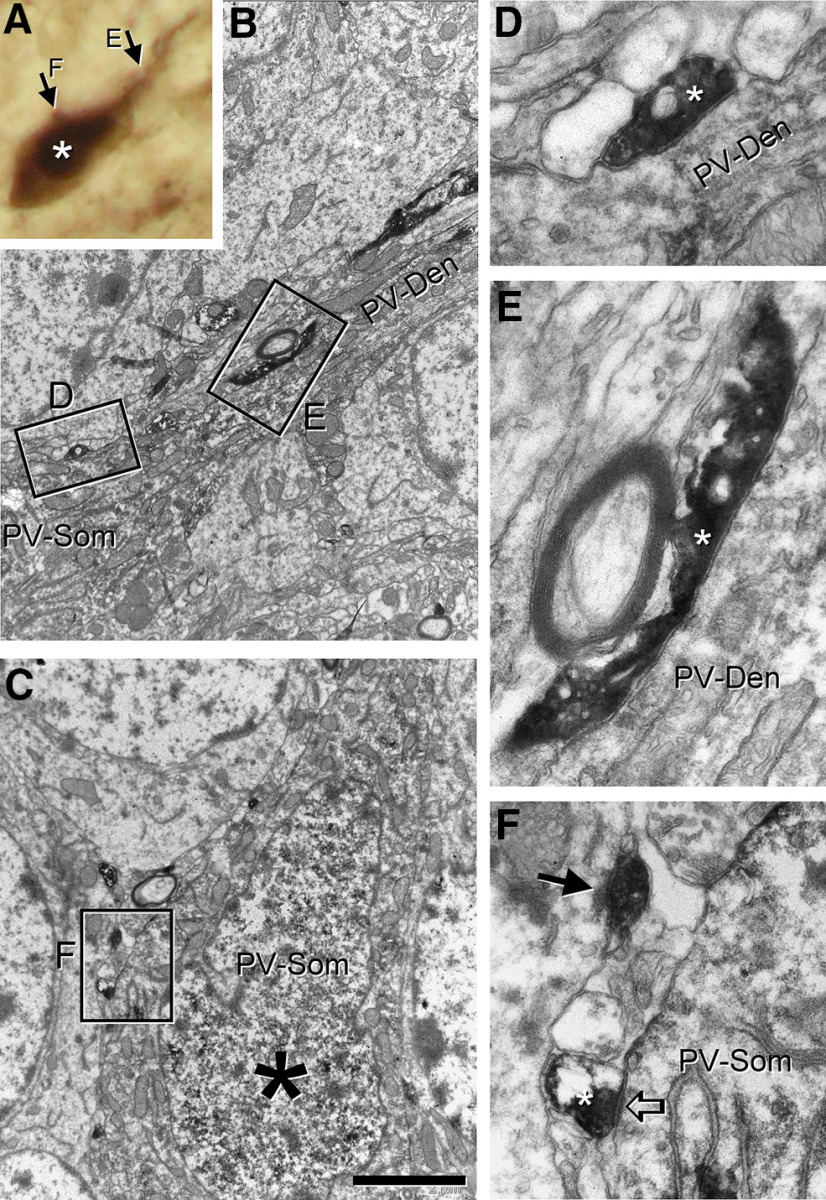
Appositions from calretinin-containing mossy fiber axons on parvalbumin cells rarely make synaptic contacts when confirmed by electron microscopy. Example of a single parvalbumin cell (DAB) with apposed calretinin fibers (DAB-Ni) studied by correlative light and electron microscopy. ***A***, Soma and apical dendrite of the parvalbumin cell (asterisk) under light microscopy apparently contacted by calretinin fibers. The boutons on the cell (arrows) were analyzed for connectivity by electron microscopy (see the corresponding images indicated by letters). ***B***, ***C***, Low-magnification electron microscopic images taken of the proximal dendritic segment (***B***) and soma (***C***, asterisk) of the cell. The boxes contain calretinin elements apposed to the cell that are shown enlarged in ***D–F***. ***D–F***, Calretinin elements tested for contacts with the parvalbumin cell were analyzed in all consecutive sections. Calretinin varicosities (asterisks) often did not establish synaptic specializations (***D***, ***E***), however, a few of them (***F***, open arrow) presented a small postsynaptic density and synaptic cleft, although the asymmetric nature of the contact is dubious. As a reference, the asymmetrical contact is clearly seen on a neighboring soma shown in ***F*** (arrow). PV-Den, parvalbumin dendrite; PV-Som, parvalbumin soma. Scale bar: 2 μm (***B***, ***C***) and 400 nm (***D–F***).

To confirm the data of the PSD95 experiment, we analyzed the ultrastructure of calretitin-expressing boutons in double immunostained sections using electron microscopy. In this set of experiments, calretinin and parvalbumin were visualized by DAB-Ni and DAB staining, respectively. This methodology allowed us to distinguish profiles stained at the light microscopic level and then perform correlative light and electron microscopy. A total number of eight parvalbumin cells were analyzed with this method. Only parvalbumin interneurons having evidence of calretinin-positive elements in apposition at the light microscopic level were studied. These cells were analyzed in serial ultrathin sections up to the depth that antibody penetration allowed. We found that calretinin-positive elements in apposition to parvalbumin cells in the granule cell layer rarely (4.8%, three out of 62) made asymmetric synaptic contacts on parvalbumin cells in the granule cell layer (Fig. 3). In molecular layer interneurons, however, the frequency of calretinin-positive boutons in apposition to parvalbumin profiles was higher (11.1%, six out of 54). In all cases, however, calretinin-positive boutons making asymmetrical contacts were found on the parvalbumin dendrites in the inner molecular layer.

Many of the calretinin-positive elements corresponded to thin profiles, running along parvalbumin interneurons in the granule cell layer (Fig. 3*A*,*B*). The proximity of these fibers made us suppose that the probability of presenting synaptic contacts with the soma and apical dendrite was rather high. In contrast, no clear postsynaptic membrane specialization supporting excitatory neurotransmission was observed. We sometimes found a submembranous density under a “climbing” calretinin-positive fiber that could suggest a synaptic contact, but no other features of synaptic boutons appeared, such as nearby mitochondria, synaptic vesicles or an enlargement of the fiber. Electron density in the DAB-Ni-labeled calretinin element prevented us to discard them as puncta adherentia junctions. Finally, calretinin-containing boutons establishing asymmetric synaptic contacts with non-parvalbumin elements were also observed in the near vicinity of parvalbumin-positive cells (Fig. 3*F*, arrow).

**Figure 4.**
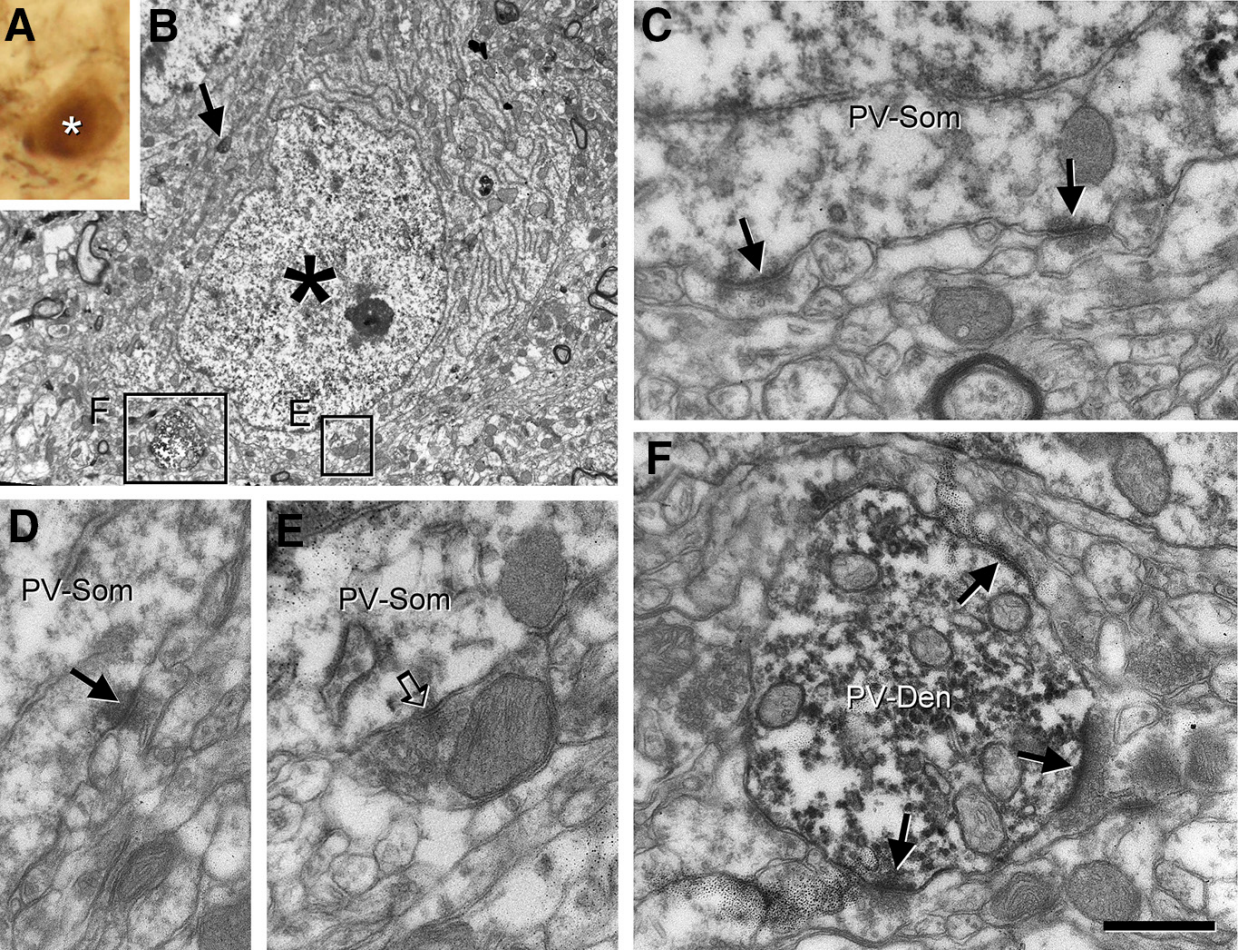
Most contacts on the perisomatic region of parvalbumin interneurons are not from mossy cells. Example of a single parvalbumin cell (DAB) with apposed calretinin fibers (DAB-Ni) studied by correlative light and electron microscopy. ***A***, Soma of the parvalbumin cell (asterisk) under light microscopy apparently contacted by calretinin fibers. ***B***, Low-magnification electron microscopic image of this soma (asterisk). A mossy cell bouton can be seen apposed to the plasma membrane (arrow), but did not form a synaptic contact. The boxes contain other presynaptic elements apposed to the cell that are shown enlarged in ***E***, ***F***. ***C***, ***D***, The soma was contacted by numerous small boutons filled with round clear vesicles making large asymmetric synapses (arrows). These boutons were not immunoreactive for calretinin. ***E***, A bouton visualized by DAB forms a symmetric synapse on the parvalbumin cell (open arrow) and shows the hallmarks of parvalbumin basket cell boutons with clear pleomorphic vesicles and large mitochondria. ***F***, A hilar juxtagranular parvalbumin dendrite (likely belonging to other cell based on its higher level of immunoreactivity) is covered by several non-immunoreactive small boutons making large asymmetrical synaptic contacts (arrows) similar to the soma analyzed. PV-Den, parvalbumin dendrite; PV-Som, parvalbumin soma. Scale bar: 2 μm (***B***, ***C***) and 400 nm (***D–F***).

On the other hand, there were numerous boutons unlabeled for calretinin establishing asymmetric synapses on parvalbumin-positive cells (Fig. 4). The morphology of unlabeled excitatory contacts resembled the en passant boutons from mossy fibers described by Blasco-Ibáñez et al. (2000; Fig. 4*C*,*D*). They were rather small and had round vesicles, but dense core vesicles could be also observed. They established large asymmetric synaptic contacts on the postsynaptic parvalbumin profiles. Parvalbumin dendrites in the juxtagranular hilus were likewise covered by boutons unlabeled for calretinin, making asymmetric contacts with the same morphology as on the soma (Fig. 4*F*).

In conclusion, although a total of nine calretinin boutons were found to establish asymmetric synaptic contacts on the perisomatic region of parvalbumin cells, this innervation was indeed scarce. In addition, under our conditions, no clear difference was observed for parvalbumin interneurons with distinct morphology except for one type: the molecular layer parvalbumin-positive interneuron, which received a somewhat larger amount of synaptic contacts from calretinin boutons on their perisomatic region. These data suggest that the presence of calretinin boutons establishing asymmetric synapses on parvalbumin elements depends mainly on the location of the somata and dendrites. As the parvalbumin cells receiving more calretinin inputs were present in the inner molecular layer, but rarely in the granule cell layer, these data point to a layer specificity rather than to a cell topology.

### Supramammillary afferents do not participate in the perisomatic excitatory innervation of parvalbumin interneurons

**Figure 5.**
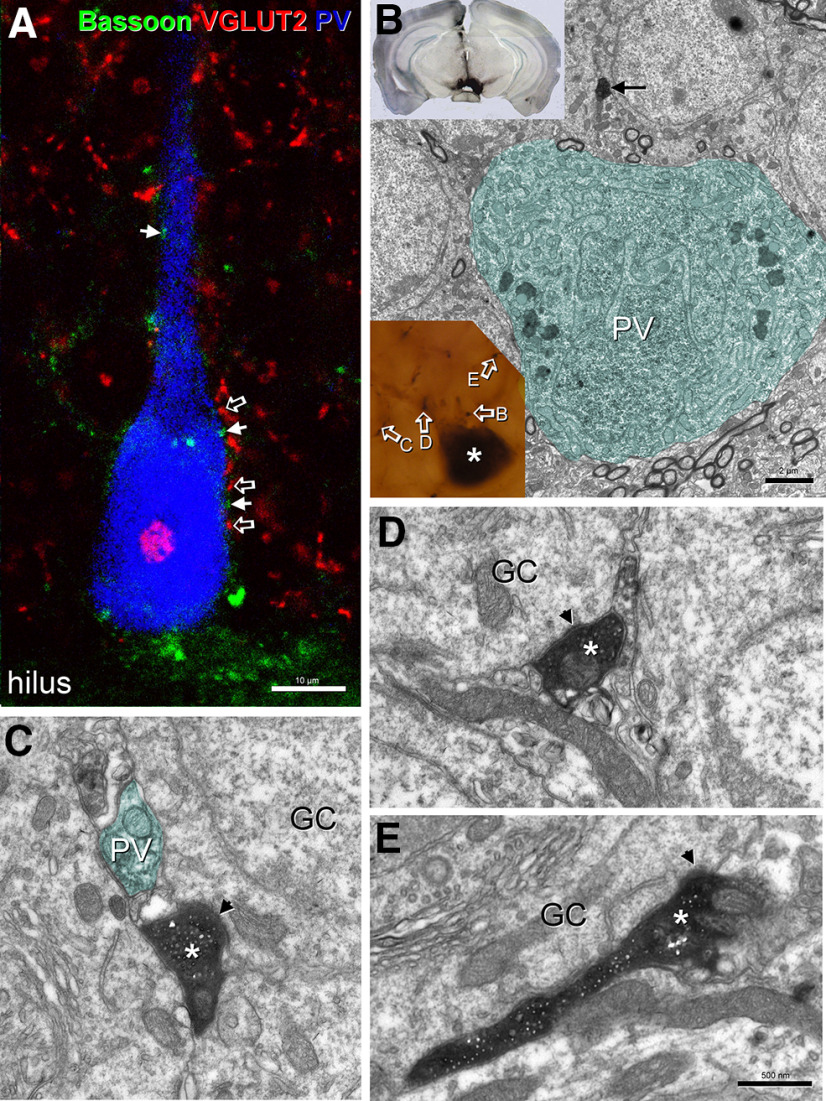
Fibers from the supramammillary nucleus do not contact soma and proximal dendrites of parvalbumin interneurons. We studied the fibers from the supramammillary nucleus using either VGLUT2 as a marker or anterograde labeling using BDA 10 kDa. ***A***, Triple immunostaining for VGLUT2 (red), bassoon (green), and parvalbumin (blue) failed to show clear boutons of subcortical origin (open arrows) with presynaptic release sites (arrows) apposing parvalbumin interneurons located in the granule cell of the dentate gyrus. ***B***, Using anterograde labeling with BDA 10 kDa from supramammillary nucleus (inset for injection site), we selected putative candidates that presented fiber apposition to parvalbumin cells (asterisk) in the dentate gyrus (inset), followed by analyzing the boutons using electron microscopy to test whether the boutons contacted the cells. Open arrows label some of the boutons from which electron micrographs are shown. Parvalbumin-expressing structures have been colored for easy identification. Bouton in panel ***B*** (arrow) approached the parvalbumin cell, but did not contact it. ***C–E***, Three different boutons were followed through the sections until synaptic contacts formed by them were identified. In all three cases, the axon terminals (asterisks) made asymmetric synapses only with granule cell somata (arrows). gcl, granule cell layer; GC, granule cell; PV, parvalbumin. Scale bar: 10 μm (***A***), 2 μm (***B***), and 500 nm (***C***, ***D***).

In addition to the mossy cell innervation, supramammillary afferents that preferentially terminate in the inner molecular layer with some collaterals entering into the granule cell layer (Maglóczky et al., 1994) may also contact parvalbumin interneurons. By examining immunostained sections for vesicular glutamate transporter 2 (VGLUT2) as a marker for this subcortical input (Boulland et al., 2009), and bassoon as marker for presynaptic release sites, we could not observe that VGLUT2-containing elements apposing parvalbumin cells express bassoon (Fig. 5*A*). Therefore, we conclude that supramammillary afferents do not make contact on the perisomatic region of parvalbumin cells. To strengthen this surprising observation, we combined anterograde labeling of afferents from the supramammillary nucleus using BDA with parvalbumin immunostaining. Despite selecting cells with fibers that seemed to appose the parvalbumin cell on their soma or proximal dendrites, we failed to find synaptic contacts on these interneurons using electron microscopy, although many times they were next to parvalbumin elements (Fig. 5*C*). The BDA fibers made scarce contacts in this area and always on granule cell somata (Fig. 5*D*,*E*), in agreement with previous findings (Maglóczky et al., 1994).

Therefore, although we cannot rule out that some parvalbumin interneurons in the dentate gyrus are contacted by this subcortical projection as it has been observed in monkeys (Leranth and Nitsch, 1994; Nitsch and Leranth, 1994), our data suggest that supramammillary input is not a substantial source of perisomatic innervation for the majority of fast-spiking interneurons, at least in mice.

### Perisomatic excitatory innervation from Timm-positive boutons on parvalbumin interneurons in the granule cell layer

**Figure 6.**
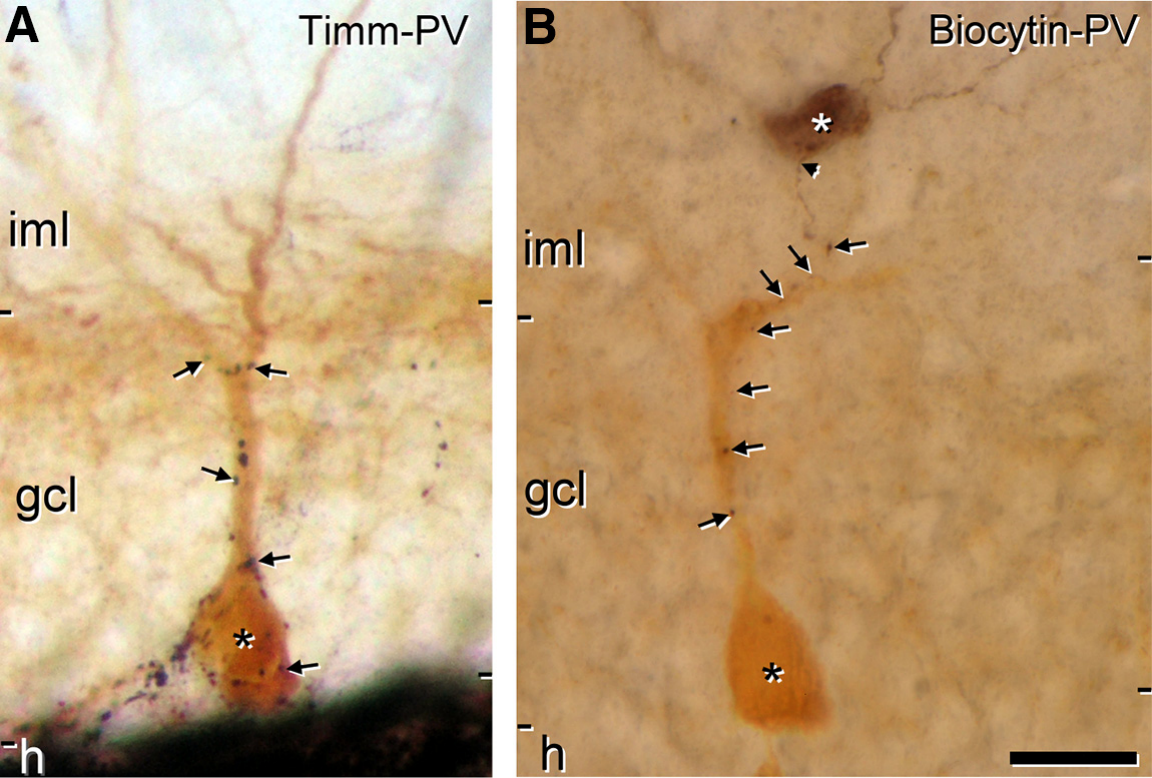
SGCs contacting parvalbumin interneurons resemble the innervation by Timm-stained boutons. ***A***, A parvalbumin cell (asterisk) in the granule cell layer contacted by Timm-stained boutons (arrows). Because of the nature of the Timm staining only the axonal varicosities are visible and the origin of them cannot be resolved. The image was reconstructed from several optical planes. ***B***, An intracellularly filled SGC (white asterisk) in the inner molecular layer gives rise to an axon (arrowhead) that descends in apposition to a parvalbumin cell (black asterisk) in its way to the hilus. The axonal varicosities (arrows) formed close apposition with the surface of the parvalbumin cell. gcl, granule cell layer; h, hilus; iml, inner molecular layer; oml, outer molecular layer. Scale bar: 20 μm.

Timm-positive boutons provide a major source of perisomatic innervation onto parvalbumin interneurons (Ribak and Peterson, 1991; Kneisler and Dingledine, 1995; Blasco-Ibáñez et al., 2000). In several of these studies, Timm staining has been used as an evidence of this innervation originating from granule cells, as their axon collaterals, the mossy fibers have vesicular zinc in their synaptic boutons at a high concentration. However, these studies were performed in the rat. To confirm the presence of this innervation in mice, we combined the Timm technique with parvalbumin immunostaining in two adult mice. In the mouse, similarly to the rat, Timm-positive boutons formed basket-like arrangements surrounding the parvalbumin-positive interneurons in the granule cell layer, and followed closely the shape of the dendrites present in this area (Fig. 6*A*). We confirmed using electron microscopy that these boutons make asymmetric synaptic contacts on the parvalbumin cells (data not shown). They were smaller than the mossy terminals characteristic of the mossy fibers, had round clear vesicles and presented dense core vesicles (Blasco-Ibáñez et al., 2000).

### Typical granule cells do not have axon collaterals in the granule cell layer

To identify the source of the Timm-positive innervation on the parvalbumin interneurons, we examined the morphology of individual granule cells by intracellular labeling them with biocytin in acute brain slices. This procedure allows for complete analysis of the dendritic and axonal arbor present in the slice.

A total of 19 granule cells located within the granule cell layer were evaluated for this objective. Generally, cells located in the subgranular zone were excluded from intracellular filling to reduce the possibilities of filling immature granule cells. In accordance with previously published data (Acsády et al., 1998; Frotscher et al., 2006), in our sample of granule cells, no collaterals were found in the granule cell layer, and no varicosities were observed until the axon reached the hilus. In addition, we did not find any mossy fiber collaterals raising from the hilus and entering the granule cell layer. Thus, it is highly unlikely that typical granule cells innervate the perisomatic region of parvalbumin interneurons.

### SGCs

Our unexpected results showing that none of the potential sources, i.e., mossy cells, typical granule cells and supramammillary afferents, provided significant perisomatic glutamatergic inputs onto parvalbumin interneurons, prompted us to test whether a scarce type of granule cells, the SGCs may contribute to this innervation. SGCs were intracellularly filled in slice preparations similarly to typical granule cells.

**Figure 7.**
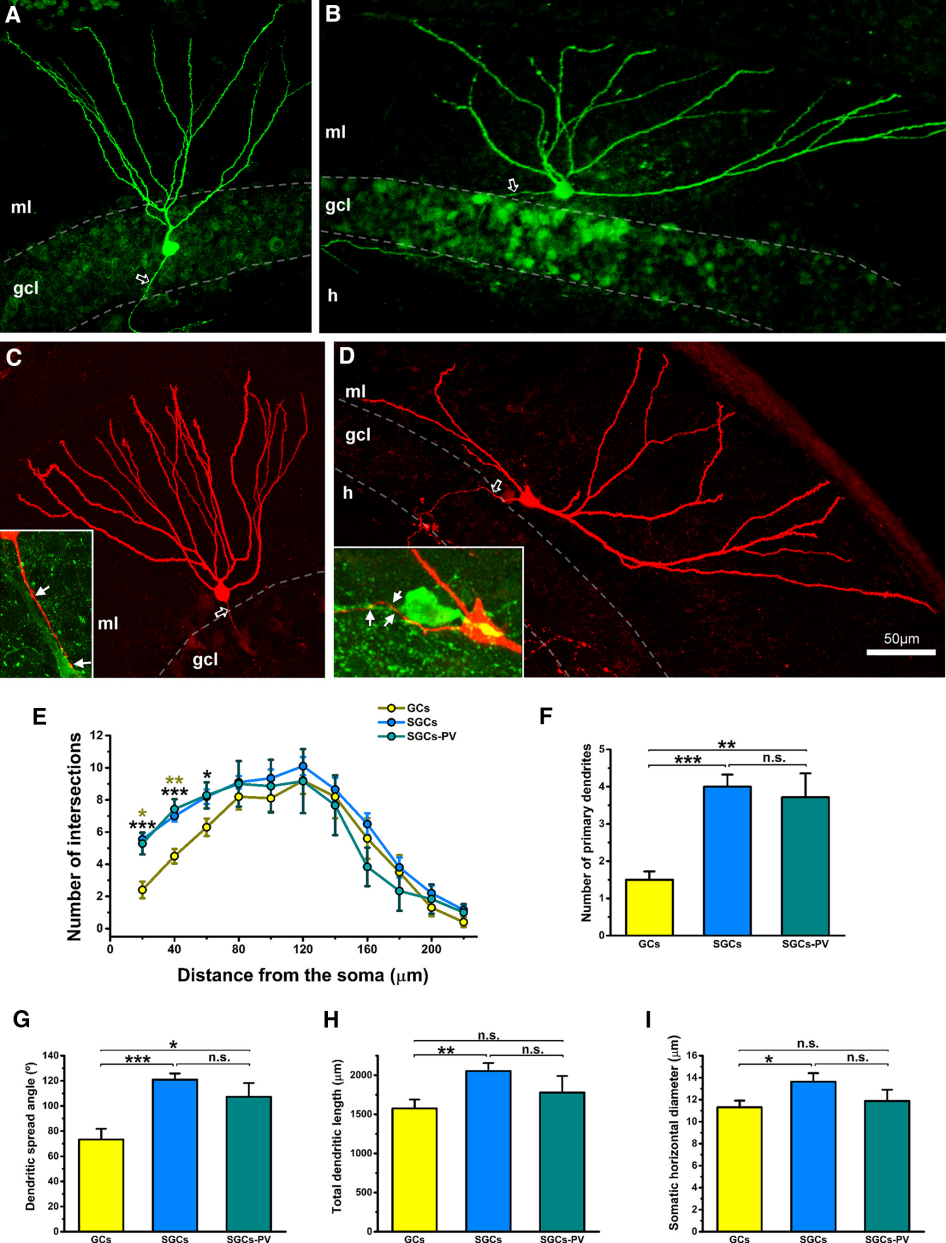
The morphology of SGCs differs from normal granule cells. ***A***, Specimen of normal granule cell filled with biocytin. One dendrite arises from the soma, the axon arises vertically and goes directly toward the hilus (arrow). ***B***, Example of SGC. Several dendrites arise from the soma, whereas the axon arises horizontally and enters the granule cell layer after running in the inner molecular layer (arrow). ***C***, ***D***, Example of the morphology of two semilunar cells with axons (open arrows) in apposition to parvalbumin interneurons. Insets show some of the biocytin-filled boutons (arrows) in close apposition to parvalbumin profiles. ***E–I***, Morphologic analysis shows that SGCs, including those that make contacts onto parvalbumin interneurons in the granule cell layer, give rise to more dendrites close to the soma as shown with the Sholl analysis (***E***, black symbols label differences between granule cells and SGCs, khaki symbols label significance between granule cells and semilunar cells innervating parvalbumin interneurons), a larger number of primary dendrites (***F***), and a wider spread of the dendritic arbor (***G***) than granule cells. SGCs also have a larger total dendritic length (***H***) and larger somatic horizontal diameter (***I***) than granule cells, though this difference was not statistically different for semilunar cells innervating parvalbumin interneurons. Asterisks show significance of the statistical analysis; *p < 0.05, **p < 0.01, ***p < 0.001; gcl, granule cell layer; h, hilus; iml, inner molecular layer; ml, molecular layer; n.s., not significant. Scale bar: 50 μm.

According to previous studies (Williams et al., 2007; Save et al., 2019), the morphologic analysis of the intracellularly labeled cells revealed that granule cells (Fig. 7*A*) and SGCs (Fig. 7*B*) could be differentiated according to their features (see Table 1). The Sholl analysis of the two types of cells (Fig. 7*E*) showed that the number of intersections up to 60 μm from the soma was statistically larger for semilunar cells than for granule cells. The number of primary dendrites was higher in semilunar cells (Fig. 7*F*), as it was the spread angle of the dendritic tree (Fig. 7*G*), the total dendritic length (Fig. 7*H*), and the horizontal diameter of the soma (Fig. 7*I*).

**Table 1:** Statistical analysis of the morphologic characteristics of normal granule cells, SGCs, and SGCs contacting dentate parvalbumin interneurons

	GCs	SGCs	SGC-PVs	MW (GCs vs SGCs)	MW (GCs vs SGCs-PV)	MW (SGCs vs SGCs-PV)
Number of primary dendrites	1.5 ± 0.22 (*n* = 10)	4.0 ± 0.32 (*n* = 18)	3.75 ± 0.56 (*n* = 8)	*p* < 0.001	*p* = 0.003	*p* = 0.797
Dendritic spread angle (°)	73.4 ± 8.5 (*n* = 10)	120.9 ± 5.0 (*n* = 18)	107.3 ± 11.0 (*n* = 8)	*p* < 0.001	*p* = 0.037	*p* = 0.389
Total dendritic length (μm)	1575 ± 114 (*n* = 10)	2055 ± 103 (*n* = 20)	1779 ± 212 (*n* = 4)	*p* = 0.009	*p* = 0.358	*p* = 0.296
Somatic horizontal diameter (μm)	11.3 ± 0.59 (*n* = 10)	13.6 ± 0.79 (*n* = 17)	11.9 ± 1.03 (*n* = 8)	*p* = 0.039	*p* = 0.894	*p* = 0.210
Sholl analysis						
Intersections at 20 μm	2.4 ± 0.52 (*n* = 10)	5.55 ± 0.38 (*n* = 20)	5.29 ± 0.68 (*n* = 7)	*p* < 0.001	*p* = 0.010	*p* = 0.865
Intersections at 40 μm	4.5 ± 0.45 (*n* = 10)	7.0 ± 0.33	7.43 ± 0.61 (*n* = 7)	*p* < 0.001	*p* = 0.005	*p* = 0.571
Intersections at 60 μm	6.3 ± 0.54 (*n* = 10)	8.2 ± 0.45 (*n* = 20)	8.29 ± 0.80 (*n* = 7)	*p* = 0.022	*p* = 0.068	*p* = 0.845
Intersections at 80 μm	8.2 ± 0.80 (*n* = 10)	9.1 ± 0.39 (*n* = 20)	9.0 ± 1.41 (*n* = 7)	*p* = 0.392	*p* = 0.730	*p* = 0.843
Intersections at 100 μm	8.1 ± 0.82 (*n* = 10)	9.35 ± 0.54 (*n* = 20)	8.86 ± 1.64 (*n* = 7)	*p* = 0.436	*p* = 0.883	*p* = 0.285
Intersections at 120 μm	9.2 ± 0.83 (*n* = 10)	10.1 ± 0.57 (*n* = 20)	9.17 ± 1.99 (*n* = 6)	*p* = 0.463	*p* = 0.620	*p* = 0.104
Intersections at 140 μm	8.2 ± 1.33 (*n* = 10)	8.65 ± 0.73 (*n* = 20)	7.67 ± 1.86 (*n* = 6)	*p* = 0.773	*p* = 0.785	*p* = 0.409
Intersections at 160 μm	5.6 ± 1.27 (*n* = 10)	6.5 ± 0.67 (*n* = 20)	3.83 ± 1.19 (*n* = 6)	*p* = 0.506	*p* = 0.412	*p* = 0.061
Intersections at 180 μm	3.5 ± 1.06 (*n* = 10)	3.8 ± 0.61 (*n* = 20)	2.33 ± 1.23 (*n* = 6)	*p* = 0.876	*p* = 0.532	*p* = 0.268
Intersections at 200 μm	1.3 ± 0.54 (*n* = 10)	2.2 ± 0.47 (*n* = 20)	1.83 ± 0.91 (*n* = 6)	*p* = 0.283	*p* = 0.727	*p* = 0.683
Intersections at 220 μm	0.4 ± 0.31 (*n* = 10)	1.2 ± 0.28 (*n* = 20)	1.0 ± 0.51 (*n* = 6)	*p* = 0.087	*p* = 0.261	*p* = 0.846

Data are shown as mean ± SEM. For the comparison of the results obtained by morphometric analysis, the Mann–Whitney test was used.

To evaluate whether SGCs are a source of excitatory input to parvalbumin interneurons, a first batch of 41 SGCs was filled with biocytin, re-sectioned, and developed with DAB-Ni. Only cells that were located in the inner molecular layer close to the granule cell layer and which had a typical semilunar morphology were used for the subsequent parvalbumin immunostaining. From these cells, four presented putative contacts on parvalbumin interneurons that clearly resembled those of Timm-positive fibers (Fig. 6).

Additionally, we intracellularly filled a second batch of SGCs that were visualized with streptavidin-Cy3. In this batch, slices were immunostained for parvalbumin without re-sectioning and possible contacts were checked using confocal microscopy. From this second batch, four out of 47 cells presented putative contacts on the perisomatic region of parvalbumin interneurons located in the granule cell layer.

We pooled the eight inner molecular layer granule cells with axonal collaterals making contacts on parvalbumin cells in the granule cell layer (SGCs-PV) to compare them to both, granule cells and SGCs. The morphologic analysis confirmed that these cells are different from normal granule cells but cannot be distinguished from SGCs (Table 1). Sholl analysis revealed differences in the arborization between granule cells and SGC-PVs close to the soma (Fig. 7*E*), but not between semilunar cells and SGC-PVs. We also found differences in two additional morphologic parameters between granule cells and SGCs-PV in the number of primary dendrites (Fig. 7*F*) and dendritic spread angle (Fig. 7*G*), but not between semilunar cells and SGCs-PV (Fig. 7*E–I*).

**Figure 8.**
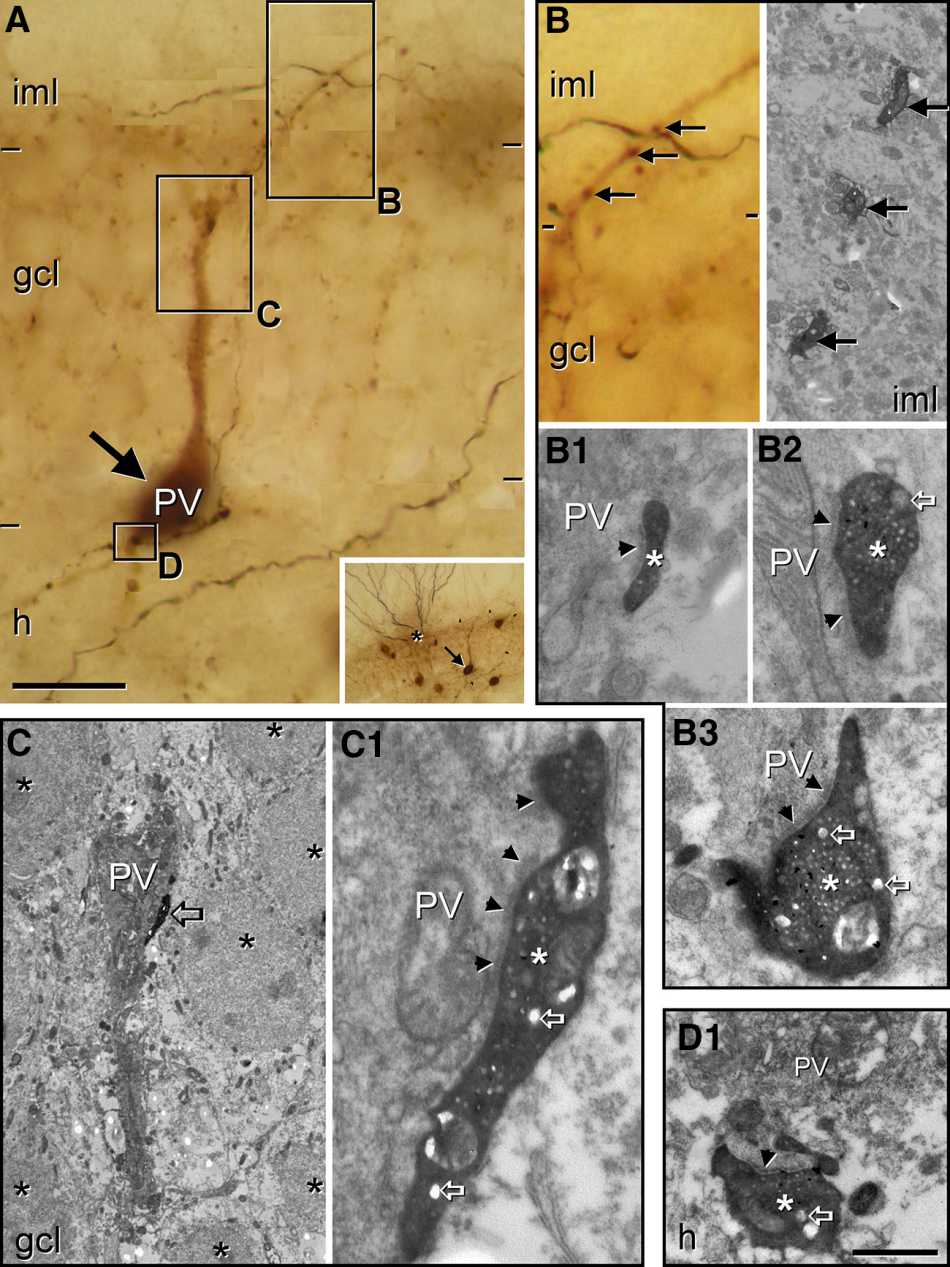
Axons of SGCs form asymmetric synapses with the perisomatic region of parvalbumin cells located in the granule cell layer. A SGC was filled with biocytin using patch-clamp technique. The section was then fixed in a fixative containing glutaraldehyde and subjected to parvalbumin immunostaining before be processed for electron microscopy. ***A***, An axon from this SGC (DAB-Ni) running in the inner molecular layer (arrowheads) sends a collateral toward the hilus delineating a parvalbumin cell labeled with DAB. In the inset, the relative position of the SGC in the inner molecular layer (asterisk) and the parvalbumin cell (arrow) is shown. The boxes show axonal varicosities of the SGC apposing the distinct portions of the parvalbumin cell in the inner molecular layer (***B***) the granule cell layer (***C***) and the hilus (***D***). ***B***, SGC axon giving rise to varicosities (arrows) along a thin parvalbumin dendrite originating from the parvalbumin cell in the inner molecular layer (left panel). The same string of boutons is presented on an electron micrograph shown in the right panel. ***B1–B3***, Higher magnification of boutons shown in ***B*** at the level where they form large asymmetric synapses on the parvalbumin dendrite (arrowheads). The boutons are filled with round vesicles as well as many dense core vesicles (open arrows). ***C***, A correlative electron microscopic image taken from the granule cell layer (asterisks label granule cells) shown in ***A***. A large bouton from the SGC (open arrow) contacting the parvalbumin cell. ***C1***, This large bouton (asterisk) makes an asymmetric synapse (arrowheads) on the parvalbumin cell and is filled with round vesicles and dense core vesicles (open arrows). ***D1***, Bouton (asterisk) from the SGC in the juxtagranular hilus makes asymmetric synapse on a proximal dendrite of the parvalbumin interneuron (arrowhead). Complementary results are presented in Extended Data Figure 8-1. gcl, granule cell layer; h, hilus; iml, inner molecular layer; oml, outer molecular layer. Scale bar: 20 μm (***A***) and 500 nm (***B–D***).

To confirm that SGC axons did innervate granule cell layer interneurons, we searched for the synaptic contacts using electron microscopy. We selected suitable examples in which the axon contacted parvalbumin cells and penetration of antibodies as well as quality of the ultrastructure were acceptable (Fig. 8). The collaterals of the SGC axons established dendritic and perisomatic contacts on a typical parvalbumin cell (Fig. 8*A*,*B*). The electron microscopic reconstruction of the axonal collaterals in apposition to the parvalbumin cell confirmed that the fibers made asymmetric synaptic contacts on the inner molecular layer proximal dendrites of the targeted parvalbumin interneuron (Fig. 8*B*), and on the dendritic trunk of the parvalbumin interneuron in the granule cell layer (Fig. 8*C*). The boutons were small and filled with round vesicles. Their morphology was similar to the one revealed for the Timm-positive boutons on parvalbumin interneurons described earlier in rat (Blasco-Ibáñez et al., 2000). In the juxtagranular hilus, the axon also made synaptic contacts on large caliber parvalbumin dendrites probably originating from these cells (Fig. 8*D*).

10.1523/ENEURO.0323-19.2020.f8-1 Extended Data Figure 8-1 SGC establishing synaptic contacts with a parvalbumin cell have characteristics of granule cells. ***A***, The same intracellularly filled SGC as in Figure 8 visualized with DAB-Ni, while parvalbumin was developed by DAB. ***A1***, Panoramic view of the dendritic arbor of the intracellularly filled SGC (asterisk). The main axon runs along the inner molecular layer (arrows), entering into the granule cell layer and reaching the hilus, where it gives rise to collaterals and varicosities. ***A2***, Higher magnification of the dendrites of the intracellularly filled SGC shown in ***A1***. Spine morphology is similar to that of typical granule cells. ***A3***, The soma of the cell shown in ***A1***. The cell body was sitting at the border between the inner molecular layer and granule cell layer (asterisk). The axon protruding from a proximal dendrite ran along the inner molecular layer (arrow), where it could be followed in the section represented in ***A1***. ***A4***, Electron microscopy of a mossy fiber collateral originated from the SGC shown in ***A***. The fiber forms mossy boutons (asterisk) that made asymmetric synaptic contacts (arrowheads) the thorny excrescences of hilar mossy cells. ***B***, Soma and dendritic arbor of the intracellularly filled SGC whose innervation is shown in Figure 6*B*. gcl, granule cell layer; h, hilus; iml, inner molecular layer. Scale bars: 50 μm (***A1***, ***B***), 20 μm (***A2***, ***A3***), and 1 μm (***A4***). Download Figure 8-1, TIF file.

Light and electron microscopy confirmed the identity of the intracellularly labeled cells by their morphology and ultrastructure. Their dendrites were covered by spines, which received small boutons making asymmetric synaptic contacts, as expected for granule cells. The large varicosities of the axon in the hilus corresponded to typical mossy terminals that formed multiple asymmetric synapses on mossy cell complex spines (Extended Data Fig. 8-1).

In conclusion, our results showed that the Timm-positive perisomatic innervation observed on the parvalbumin cells originates mainly, if not exclusively from SGCs.

## Discussion

In the present work, we analyzed the origin and relevance of the perisomatic excitatory connectivity on the parvalbumin fast-spiking cells in the dentate gyrus.

Our first approach to reveal the contribution of the excitatory inputs to the perisomatic innervation on parvalbumin cells was to compare it with the inhibitory inputs using gephyrin and PSD95 as postsynaptic markers (Lin et al., 2004; Fritschy et al., 2008; Jackson and Nicoll, 2011). Our data showed that the perisomatic excitatory input on parvalbumin interneurons is more abundant than the inhibitory perisomatic input. This is in agreement with previous results obtained for parvalbumin interneurons in the CA1 region of the rat hippocampus (Gulyás et al., 1999). Thus, the strong excitatory drive on the soma is a common characteristic shared by all parvalbumin-expressing fast-spiking interneurons.

Dentate gyrus parvalbumin cells receive excitatory input from the entorhinal cortex via the perforant path in the outer two thirds of the molecular layer (Zipp et al., 1989), as well as from fibers of mossy cells in the inner molecular layer (Seress and Ribak, 1984). The latter study has also described a frequent perisomatic innervation from degenerated commissural fibers (originating likely from mossy cells) on parvalbumin cells present in the molecular layer, but only one synaptic contact was observed by the authors on those interneurons located in granule cell layer. As the previous study was done on degenerated boutons, their symmetric or asymmetric nature of the contacts could not be reported and therefore this innervation could originate even from hilar interneurons, which are known to project to the contralateral dentate gyrus (Zappone and Sloviter, 2001). Although it has been suggested that commissural boutons synapse on the somata of parvalbumin cells, the relevance of this projection has not been tested. These data suggest that the commissural innervation of parvalbumin cells is layer specific rather than cell type specific.

Other source of asymmetrical boutons on dentate gyrus parvalbumin cells may come from supramammillary fibers (Leranth and Nitsch, 1994; Nitsch and Leranth, 1994). However, these authors showed in monkey that most parvalbumin cells were not targeted, but only a small fraction of the parvalbumin cells located in the upper half of the granule cell layer. Supramammillary fibers co-release glutamate and GABA, but the net effect is excitatory (Hashimotodani et al., 2018). We did not find parvalbumin cells targeted by either anterograde fibers from the supramammillary nucleus nor by VGLUT2-containing axon terminals in the dentate gyrus. Therefore, we feel safe to conclude that the supramammillary nucleus does not contribute to the perisomatic excitatory drive of parvalbumin interneurons in the dentate gyrus, at least in mice.

On the other hand, it has been shown that the somata of parvalbumin cells receive a high number of Timm-positive boutons that make asymmetric contacts on them (Blasco-Ibáñez et al., 2000). Timm-positive boutons concentrate in the hilus and stratum lucidum, where they correspond to mossy fibers, the axons of granule cells. In the granule cell layer, Timm staining reveals only a few axon collaterals and, most importantly, only when a special care is taken for the detection of vesicular zinc. These collaterals have been suggested to originate from granule cells, however, no definitive proof has been provided yet, since this staining only labels the boutons and does not allow locating the cell from which they originate. Indeed, the number of granule cells is too large as compared with the number of GABAergic cells involved in these innervations (Blasco-Ibáñez et al., 2000). In addition to granule cells, other glutamatergic sources of boutons that contain zinc have been described in this area, such as the mossy cells and the axon terminals of entorhinal pyramidal cells (Pérez-Clausell and Danscher, 1985; Valente et al., 2002; Paoletti et al., 2009). There is evidence showing that entorhinal fibers may enter into the hilus through the granule cell layer (Deller et al., 1996). However, the strength of Timm staining in the axonal varicosities of these two glutamatergic neuron types is considerably lower than in the axon terminals of granule cells, yet their contribution to the Timm-stained boutons surrounding parvalbumin interneurons cannot be discarded directly.

### Perisomatic excitatory innervation from mossy cells onto parvalbumin cells in the granule cell layer

We used calretinin as a marker for mossy cell axon terminals as it has been shown that in mouse the latter are highly immunoreactive for this Ca^2+^ binding protein (Liu et al., 1996; Blasco-Ibáñez and Freund, 1997). We found, however, that varicose axons from the mossy cells that pass close to parvalbumin-positive interneurons through the granule cell layer make no contacts with them. Moreover, boutons from other cells also express calretinin in the dentate gyrus: (1) boutons coming from the supramammillary nucleus (Maglóczky et al., 1994) and (2) boutons from interneuron-specific interneurons (Gulyás et al., 1996; Hájos et al., 1996).

The possibility that some of the calretinin-positive elements that we have seen around parvalbumin interneurons come from supramammillary fibers had to be considered, since they also establish asymmetric synapses and are calretinin-immunoreactive in the rat (Maglóczky et al., 1994). However, double immunostaining for calretinin and VGLUT2 to label fibers of extracortical origin yielded no colocalization in the granule cell layer and inner molecular layer of the mouse dentate gyrus (data not shown). Calretinin boutons coming from interneurons are not abundant. The possibility of having calretinin-positive elements that establish symmetric contacts is also low, as interneuron-selective interneurons expressing calretinin avoid parvalbumin cells (Gulyás et al., 1996; Blasco-Ibáñez et al., 1998). Boutons of mossy cells and interneurons could be distinguished by combining the labeling with markers for excitatory and inhibitory postsynaptic sites (PSD95 and gephyrin, respectively) or by electron microscopy. Therefore, the calretinin boutons apposing parvalbumin cells are very likely to come from the hilar mossy cells. In our correlated light and electron microscopic study, no clear synaptic contacts from calretinin-containing mossy cell boutons could be found on the perisomatic region of parvalbumin cells with soma located in the granule cell layer. We found, however, synaptic contacts from calretinin boutons on parvalbumin cells and dendrites in the inner molecular layer. In summary, our results show that the main, and almost exclusive, source of perisomatic excitatory innervation onto parvalbumin-positive cells in the granule cell layer are the Timm-positive boutons. In line with this suggestion, excitatory synapses that shared the characteristics of en passant boutons from Timm-positive fibers were easily found. These results collectively indicate that mossy cells do not provide a substantial perisomatic excitatory drive onto parvalbumin cells in the granule cell layer. Importantly, mossy cell innervation seems to be layer specific rather than cell type specific in comparison to the projection from the entorhinal cortex (Zhao et al., 2003). Thus, a different ratio of perisomatic excitatory innervation from mossy cells may be present on parvalbumin cells whose soma is located in the basal part of the granule cell layer respective to those located in the apical part and inner molecular layer.

### Origin of the Timm-positive boutons on parvalbumin somata in the granule cell layer

Previous studies have shown that parvalbumin interneurons are innervated by Timm-positive fibers, which contain high levels of Zn^2+^ (Ribak and Peterson, 1991; Blasco-Ibáñez et al., 2000; Seress et al., 2001; Frotscher et al., 2006). These particular Timm-positive fibers have been presumed to arise from granule cells. Indeed, the ultrastructural features of these boutons are similar to those of mossy fibers contacting hilar interneurons, with small round synaptic vesicles and a few dense core vesicles (Claiborne et al., 1986; Acsády et al., 1998), although their postsynaptic partner are not spines, either simple or complex. As Timm-stained boutons cannot be found on the granule cell somata or in the axonal segments connecting the boutons, this prevents the identification of the cell from which they originate. On the other hand, the number of Timm-stained boutons is quite low in relation with the total number of granule cells. Therefore, even if they come from granule cells, this population of granule cells must be quite a restricted group of neurons (Blasco-Ibáñez et al., 2000). If we assume that the number of parvalbumin interneurons is in the order of 1000 in the granule cell layer of the mouse (Hernández-González et al., 2015), and approximately four to six Timm-positive fibers contact on each parvalbumin cells (even considering that all those collaterals originate from different cells), then the cell population that would generate this innervation should be limited to 4000–6000 cells. As the number of granule cells in the mouse dentate gyrus has been estimated to be around 500,000 cells (Amrein et al., 2004), approximately only 1% of granule cells would be in charge of this innervation.

### Semilunar and not typical granule cells provide the perisomatic excitatory innervation on parvalbumin interneurons located in the granule cell layer

Although granule cells in rodents have been analyzed as a homogeneous population, in recent years, SGCs have been proven to have special characteristics (Williams et al., 2007; Larimer and Strowbridge, 2010; Gupta et al., 2012). The location of these cells in the dentate gyrus makes them a suitable source for the perisomatic excitatory innervation on parvalbumin cells.

In full agreement with previous studies, where granule cells were labeled *in vitro* or *in vivo* (Acsády et al., 1998; Henze et al., 2002; Amaral et al., 2007; Zhang et al., 2012), our study confirmed that Timm-positive collaterals onto parvalbumin interneurons do not arise from typical granule cells. The granule cell axon always arose from the basal pole of the cell and projected directly through the granule cell layer to the hilus, where it ramified. In addition, no varicosities were observed on their way through the granule cell layer. In brains from epileptic animals, however, typical granule cells give rise to some axon collaterals that enter to the granule cell layer and even to the inner molecular layer (Kotti et al., 1997; Kobayashi and Buckmaster, 2003). On the contrary, in normal animals, the axonal branching pattern of the Timm-stained fibers in the granule cell layer goes from the inner molecular layer into the hilus (Blasco-Ibáñez et al., 2000).

SGCs, at least a portion of them, have axons in the inner molecular layer running parallel to the granule cell layer, where they sometimes ramify and forms one or two collaterals. (Fig. 7; Extended Data Fig. 8-1; Ramón y Cajal, 1904; Williams et al., 2007). We observed that the axons of some SGCs descend through the granule cell layer and present varicosities that form synaptic contacts with the perisomatic region of parvalbumin interneurons. However, only a part of the intracellularly labeled SGCs had axons that ramified in the granule cell layer. Several technical issues may have influenced these results: (1) incomplete filling of the axon; (2) cut axonal collaterals; and (3) dead or degenerating target parvalbumin cells in the slice that lost the antigenicity for the antibody.

One of our main findings confirmed by electron microscopy is that axons from SGCs form synaptic contacts with parvalbumin interneurons. Noteworthy, the ultrastructure of these boutons corresponds to the descriptions for the Timm-positive boutons already published (Blasco-Ibáñez et al., 2000), strongly arguing for the hypothesis that the Timm-positive innervation on parvalbumin cells mainly, if not exclusively comes from SGCs. Interestingly, boutons from the same fiber continued to form synapses on the parvalbumin dendrites present in the hilus. Innervation from Timm-positive boutons on hilar parvalbumin cells has been described previously (Seress et al., 2001).

### Relevance of our findings for the local connectivity of the dentate gyrus

Since the different excitatory inputs onto parvalbumin cells are not randomly distributed, the location of the perisomatic glutamatergic inputs could play an important role in the modulation and triggering of spiking. For any neuron, innervation located in distal dendrites is usually considered to modulate firing responses, whereas innervation in proximal dendrites, or even better at the cell body, is more important to effectively control the capability of the cell to fire somatic action potentials (Cobb et al., 1995; Miles et al., 1996; Veres et al., 2017).

Perisomatic excitatory drive of parvalbumin interneurons provided by local principal cells has been previously studied in different brain areas such as the visual cortex (Buhl et al., 1997), the hippocampus and the amygdala (Sik et al., 1993; McDonald et al., 2005; Andrási et al., 2017) as well as in the dentate gyrus in both control and epileptic animals (Ribak and Peterson, 1991; Kotti et al., 1997; Blasco-Ibáñez et al., 2000). Furthermore, parvalbumin cells in the hilus and CA3 also receive Timm-positive boutons (Seress et al., 2001), similarly to those parvalbumin interneurons that are located in the granule cell layer. The presence of this perisomatic glutamatergic input in all cortical structures studied suggests a general control mechanism of fast-spiking interneuron excitability.

**Figure 9.**
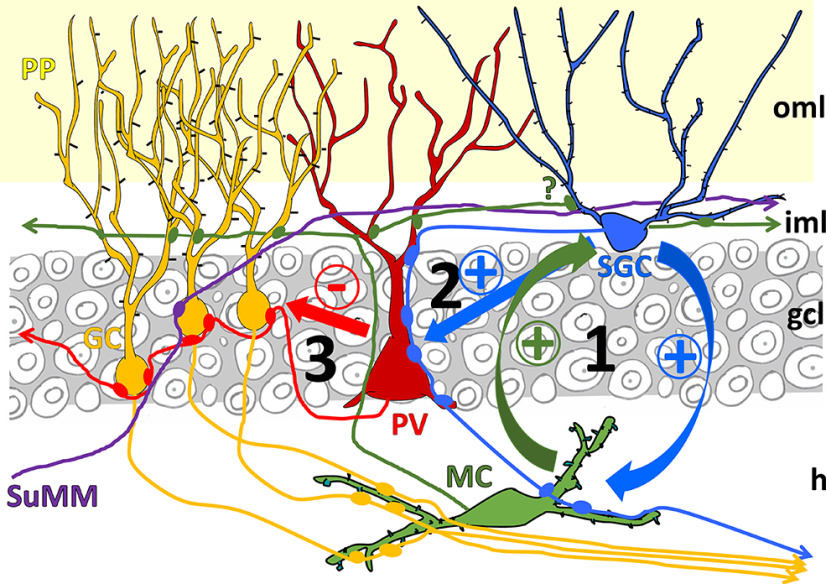
Diagram of the connectivity involving the excitatory perisomatic innervation of dentate gyrus parvalbumin interneurons. Dentate gyrus parvalbumin interneurons (PV) receive perisomatic excitatory input mainly from SGCs. Input from mossy cells is rare in the granule cell layer (gcl) but occurs in the inner molecular layer (iml). Supramammillary fibers (SuMM) do not seem to contact parvalbumin cells on the soma. When SGCs fire, they could activate mossy cells with which they engage in repeating firing known as hilar up states (1), at the same time SGCs will excite parvalbumin interneurons (2) that would control large populations of granule cells (3) synchronizing their firing when excited by the perforant pathway. At the same time, mossy cells likely project back to SGCs, although this projection has not been specifically described (question mark). GC, granule cells; h, hilus; MC, mossy cells; oml, outer molecular layer; PP, perforant pathway; PV, parvalbumin interneurons; SuMM, projection from the supramammillary nucleus.

The specific innervation of dentate parvalbumin interneurons by SGCs places this granule cell type into a privileged position in controlling the activity of neural ensembles in the dentate gyrus (Fig. 9). SGCs are engaged during hilar up-states together with the mossy cells (Larimer and Strowbridge, 2010). Thus, during this special network state, SGCs may effectively and simultaneously discharge on both mossy cells and parvalbumin cells, causing parallel excitation of the granule cell dendrites in the inner molecular layer and inhibition of the perisomatic region of granule cells. Recent results obtained *in vivo* show that granule cell firing is quite sparse in different environments, whereas mossy cells firing is robust (GoodSmith et al., 2017; Senzai and Buzsáki, 2017), which may be explained, at least partially by our structural data. We propose that SGCs, although they are few in number, are key circuit elements in the dentate function. As the SGCs are able to drive simultaneously the firing of mossy hilar cells and parvalbumin interneurons in a feed-forward manner, during this temporal window strong entorhinal excitatory input can discharge only a restricted granule cell population by overcoming their perisomatic inhibition with the help of mossy cell excitation. The sparse firing of granule cells is suggested to be a critical factor for pattern separation that is considered as the main computation function of the dentate gyrus in the hippocampal circuitry (Nakazawa, 2017).

## Synthesis

Reviewing Editor: Katalin Toth, Universite Laval Decisions are customarily a result of the Reviewing Editor and the peer reviewers coming together and discussing their recommendations until a consensus is reached. When revisions are invited, a fact-based synthesis statement explaining their decision and outlining what is needed to prepare a revision will be listed below. The following reviewer(s) agreed to reveal their identity: Floris Wouterlood.

The current study provides valuable and novel information. The study is generally carefully executed but in many aspect it lacks the necessary quantitative assessment. There are two major issues the authors need to address:

1. Morphometric differences between granule cells and semilunar granule cells. The authors should provide more quantitative data on how they differentiated between these cells types and support their analysis with reconstructions and/or images.

2. Validation of mamillary body injection sites.

Both reviewers made several other constructive comments, the authors should carefully consider these points in their reply.

Rev. #1.

The authors have applied a spectrum of neuroanatomical end neurophysiological techniques, combined with electron microscopy, to extract information on the innervation of parvalbumin neurons in the granular layer of the mouse dentate gyrus. I perceive their work as very well designed and well performed. They throw all available methods into battle, to speak in military terms.

The final conclusion from their work is that the semilunar granule cells are suspected of driving the parvalbumin neurons, not the mossy cells, perforant pathway input or supramammillary neurons. An additional granule cell - interneuron control system to keep the majority of granule cells in check?

Ar various spots sentences could be written more condensed, and in addition there are several omissions and small errors, however first a conceptual remark/question:

The authors write in Materials and Methods:

‘.....Only those puncta that were in close apposition to the edge of the parvalbumin profile with no apparent space between both profiles, were considered as positive.’

At this point in Figure 1D there are gephyrin and PSD punctae INSIDE the PV cell body and dendrite - most obviously PSD punctae. Because both PSD and gephyrin are located postsynaptically I would expect them to be inside the PV cell, not to appose the cell. Basically any apposition of a punctum and PV (on the outer face of the PV cells limiting membrane (‘outside decoration’) should be considered negative, only the punctae colocalized on the inside of the limiting membrane of the PV neuron should count positive! (‘inside decoration’) (!)

Crucial here is how you define ‘edge’ or the pixel-exact position of the limiting membrane envelope of a neuron. Given a Z step size of 500 nm the chance of observing a razor sharp ‘edge’ is pretty close to or equal to zero. Consider outer membranes of parvalbumin cells as balloons, bags, curved surface sheets. This implies that, with a given optical thickness of a section, you observe most of the time an oblique, wedge-type, sloped external face of the outer cell membrane. Because parvalbumin is inside the cell and immediately adjacent to the outer cell membrane, there should be color mixing (colocalization) of parvalbumin signal and the PSD or gephyrin signal, thanks to the postsynaptic location of both markers.

Indeed a lot of punctae can be seen in Fig 1B inside the Pv dendrite. Is Fig 1B a Z-projection image?

With synaptophysin one should be aware that contrary to PSD and gephyrin this is a synaptic vesicle marker and not a synapse marker. Inside a synaptic bouton there is a cluster of synaptic vesicles. The synaptic vesicles usually, but not always, tend to converge to the presynaptic membrane. Apposition of a synaptophysin punctum and a PV cell only proves that they are close to each other, not that there is necessarily a synapse. The opposite is true as well: if there is no apposition then there is no substrate for a synapse.

Be aware that ANY image that you acquire at 63x primary magnification is always blurred (Abbe diffraction). Did you apply deblurring or deconvolution? (not some undocumented plugin or proprietary thing but statistically sound deconvolution algorithms?

In conclusion: My estimation of your manuscript is: very well done, good question, nice experiments, smart and systematical combination of tools. The manuscript is because of this somewhat lengthy, with numerous awkward phrasing. Please add a scheme (Figure) indicating cellular relationships. Provide details about your confocal microscope’s settings, improve the section describing appositions, add a note on statistics, correct typos and you’re done as far as I am concerned. Good luck!

=================================

some remarks on the use of English

Abstract:

p2, line 5: ‘scare’ must be ‘scarce’

p. 12‘......PSD95 is located in the postsynaptic density at the asymmetric synapses and functions as...’ - remove ‘the’ so that

‘...PSD95 is located in the postsynaptic density at asymmetric synapses and functions as...’

p 16. ‘excitatory postsynaptic specialization’. I think that this is an awkward expression. The specialization does not excite. I would prefer ‘postsynaptic membrane specialization supporting excitatory neurotransmission’

p. 16. The sentence including ‘.......with distinct morphology except for one type: the molecular layer parvalbumin-

positive interneurons, which received somewhat larger amount of synaptic contacts from

calretinin boutons on their perisomatic region....’ mixes singular and plural.

correct would be: ‘....with distinct morphology except for one type: the molecular layer parvalbumin-

positive interneurons, which received a somewhat larger amount of synaptic contacts from

calretinin boutons on their perisomatic region..’.

The next sentencve, ‘.....These data suggest that the presence of calretinin boutons establishing asymmetric synapses on parvalbumin elements depended mainly on the location of the somata and dendrites....’ Here the ‘depended’ should be present term ‘depend’.

p. 17, Interestingly the Boulland et al (2008) report unequivocally shows (in rat) colocalization of VGLUT2 and VGAT in supramammillary axon endings in the dentate gyrus

p. 17, ‘....By examining immunostained sections for vesicular glutamate transporter 2 (VGLUT2) as a maker for...’ --‘maker’ should be ‘marker’

p. 18, ‘...In some of these studies, Timm staining has been used...’ -- change ‘some’ into ‘several’

p. 22, ‘...On the other hand, it has been shown that the soma of parvalbumin cells...’ -- ‘soma’

must be ‘somata’

p. 22,‘...Therefore, it is safe to conclude, the supramammillary nucleus does not contribute..’ -- change into ‘...Therefore, we feel safe to conclude that the supramammillary nucleus does not contribute...’

p.22, the sentence ‘...In the granule cell layer, only a few axon collaterals revealed by Timm staining can be detected and, importantly,...’ can be shortened into ‘....In the granule cell layer, Timm staining reveals only a few axon

collaterals, most importantly,....’

po. 22-23, ‘...However, the strength of Timm staining in the axonal varicosities of these two glutamatergic neuron types is considerably lower than that of the granule cells...’ -- what does ‘that’ refer to (strange construction of sentence)

multiple of ‘soma’ is ‘somata’

page 25, What do you mean with 4-6000 cels: between four and six thousand or between four thousand and six thousand ?

page 25, The notation for one thousand is 1,000. five hundred thousand is 500,000

As far as supramammillary innervation concerns: A report by Boulland et al (2008) unequivocally shows (in rat) colocalization of VGLUT2 and VGAT in supramammillary axon endings in

The manuscript says: ‘For image analysis we used FIJI (ImageJ 1.49j10; Schindelin et al., 2019)’.

That single line is insufficient. FIJI is only a suite that supports a myriad of functions, algorithms and plugins. Which function(s), algorithm or plugin were applied?

There has been statistical testing (Results): PSD appositions versus gephyrin appositions on PV cells. The statistical test and test conditions are not mentioned.

Rev.#2.

In this descriptive study, the authors use immunostaining, neural tracer techniques and electron microscopy to examine the main source of somatic/perisomatic innervation of dentate parvalbumin-expressing neurons located in the dentate granule cell layer. Using triple immunostaining for parvalbumin along with PSD95 and gephyrin to identify glutamatergic and GABAergic synapses, they find that that the somata and proximal dendrites of PV neurons in the granule cell layer have more PSD95 positive synapses than gephyrin positive synapses indicating a more robust perisomatic glutamatergic innervation. By labeling for synaptic markers and calretinin, they show that mossy cell projection to PV neurons is sparse and cannot account for the robust excitatory inputs. Based on the lack of co-labeling of VGlut2+ve synapses or synaptic contacts upon anterograde labeling of the supramammillary nucleus, they find that cells from the supramammillary nucleus are unlikely to provide perisomatic glutamatergic inputs to PV neurons. However, they find that zinc localizes to perisomatic synapses on PV neurons. By examining contacts from biocytin-filled granule cells and putative semilunar granule cells and suggest that putative semilunar granule cells rather than granule cells provide perisomatic glutamatergic inputs dentate PV neurons. This is a provocative and, if validated, an interesting finding. However, the key difference between the cell types, are not quantified and no complete fills identifying the two cell types are included. Thus, in the absence of definitive classification of the cell types it is impossible to assess the validity of the conclusions.

Specific concerns

• The major concern is that the main finding hinges on discriminating between two very similar cell types and the manuscript does not include convincing morphometric or quantitative data to discriminate between the cell types. The authors need to demonstrate that the putative semilunar granule cells are indeed distinct from the classical granule cells.

• The authors indicate that they filled granule cells - however no data from these cells are presented

• Is there a sampling bias? The authors examined 16 granule cells vs. 41 putative semilunar granule cells and targeted 4 cells with molecular layer axon collaterals for further analysis

• All data are present as percentages and actual values are not included making it difficult to assess variability in the data.

• Data demonstrating that the anterograde labeling did indeed target the supramammilary nucleus is lacking. This is an important control. Additionally, VGLUT2 is not exclusive for supramammilary nucleus and can be present in septal and thalamic nuclei.

• Figures need to include quantitative data

• The statement in the discussion (pg. 21) suggests that morphologically distinct types of PV showed no differences in the proportion of perisomatic glutamatergic innervation. However, no data on cell morphologies are included, rather, PV neurons are distinguished based on somatic location. This needs to be clarified
