# Supplementary figures and images for: Semilunar Granule Cells Are the Primary Source of the Perisomatic Excitatory Innervation onto Parvalbumin-Expressing Interneurons in the Dentate Gyrus
Source: eNeuro. 2020 Jul 7;7(4):ENEURO.0323-19.2020. doi: 10.1523/ENEURO.0323-19.2020 (PMC7340841; doi:10.1523/ENEURO.0323-19.2020)

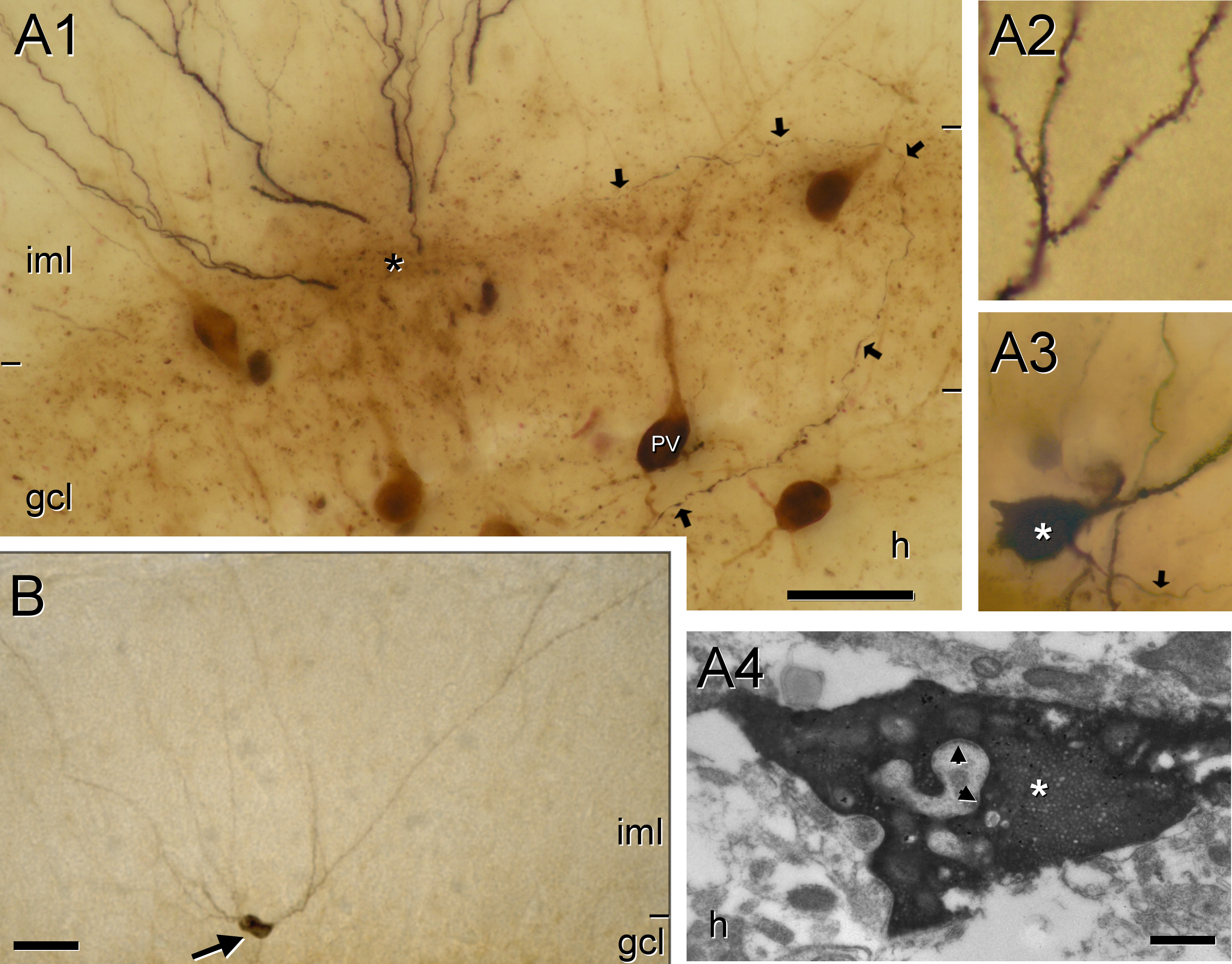

Supplement: Extended Data Figure 8-1 — SGC establishing synaptic contacts with a parvalbumin cell have characteristics of granule cells. A, The same intracellularly filled SGC as in Figure 8 visualized with DAB-Ni, while parvalbumin was developed by DAB. A1, Panoramic view of the dendritic arbor of the intracellularly filled SGC (asterisk). The main axon runs along the inner molecular layer (arrows), entering into the granule cell layer and reaching the hilus, where it gives rise to collaterals and varicosities. A2, Higher magnification of the dendrites of the intracellularly filled SGC shown in A1. Spine morphology is similar to that of typical granule cells. A3, The soma of the cell shown in A1. The cell body was sitting at the border between the inner molecular layer and granule cell layer (asterisk). The axon protruding from a proximal dendrite ran along the inner molecular layer (arrow), where it could be followed in the section represented in A1. A4, Electron microscopy of a mossy fiber collateral originated from the SGC shown in A. The fiber forms mossy boutons (asterisk) that made asymmetric synaptic contacts (arrowheads) the thorny excrescences of hilar mossy cells. B, Soma and dendritic arbor of the intracellularly filled SGC whose innervation is shown in Figure 6B. gcl, granule cell layer; h, hilus; iml, inner molecular layer. Scale bars: 50 μm (A1, B), 20 μm (A2, A3), and 1 μm (A4). Download Figure 8-1, TIF file. [file enu-eN-NWR-0323-19-s01.tif]
